# Automated Detection of Caffeinated Coffee-Induced Short-Term Effects on ECG Signals Using EMD, DWT, and WPD

**DOI:** 10.3390/nu14040885

**Published:** 2022-02-19

**Authors:** Bikash K. Pradhan, Maciej Jarzębski, Anna Gramza-Michałowska, Kunal Pal

**Affiliations:** 1 Department of Biotechnology and Medical Engineering, National Institute of Technology, Rourkela 769008, India; bikashpradhan21@gmail.com; 2 Department of Physics and Biophysics, Faculty of Food Science and Nutrition, Poznań University of Life Sciences, Wojska Polskiego 38/42, 60-637 Poznan, Poland; maciej.jarzebski@up.poznan.pl; 3 Department of Gastronomy Science and Functional Foods, Faculty of Food Science and Nutrition, Poznań University of Life Sciences, Wojska Polskiego 31, 60-624 Poznan, Poland

**Keywords:** caffeinated coffee, caffeine, the short-term effect of coffee, ECG, machine learning

## Abstract

The effect of coffee (caffeinated) on electro-cardiac activity is not yet sufficiently researched. In the current study, the occurrence of coffee-induced short-term changes in electrocardiogram (ECG) signals was examined. Further, a machine learning model that can efficiently detect coffee-induced alterations in cardiac activity is proposed. The ECG signals were decomposed using three different joint time–frequency decomposition methods: empirical mode decomposition, discrete wavelet transforms, and wavelet packet decomposition with varying decomposition parameters. Various statistical and entropy-based features were computed from the decomposed coefficients. The statistical significance of these features was computed using Wilcoxon’s signed-rank (WSR) test for significance testing. The results of the WSR tests infer a significant change in many of these parameters after the consumption of coffee (caffeinated). Further, the analysis of the frequency bands of the decomposed coefficients reveals that most of the significant change was localized in the lower frequency band (<22.5 Hz). Herein, the performance of nine machine learning models is compared and a gradient-boosted tree classifier is proposed as the best model. The results suggest that the gradient-boosted tree (GBT) model that was developed using a db2 mother wavelet at level 2 decomposition shows the highest mean classification accuracy of 78%. The outcome of the current study will open up new possibilities in detecting the effects of drugs, various food products, and alcohol on cardiac functionality.

## 1. Introduction

Caffeine is the most widely used psychoactive compound globally. A wide range of food products (e.g., tea, coffee, energy drinks, soft drinks, chocolate, etc.) contains caffeine. Coffee is the most usual form of caffeine consumption among these food products. Also, it is the most researched beverage by the scientific community [1]. The ingestion of caffeine prompts physiological and behavioral effects in the human body [2]. Many people experience an active mood and increased concentration after its consumption. Prolonged exposure to coffee/caffeine also influences various organs and organ systems. Caffeine stimulates autonomic nervous system (ANS) activities with increased catecholamine secretion. It is essential to understand the chemical nature of caffeine and its mechanism of absorption in the human body. After ingestion, caffeine gets quickly absorbed in the gastrointestinal tract [3]. Then, it moves through the cellular membrane and circulates to tissues. After the liver metabolizes the caffeine, it produces three metabolites, namely paraxanthine, theophylline, and theobromine [4,5]. The traces of these metabolites can be found in the bloodstream within 10–45 min of caffeine consumption [6,7]. Because of its excellent lipid solubility, caffeine can easily cross the blood–brain barrier and be excreted through the kidneys in due course of time. Depending upon the level of tissue uptake and the urinary clearance, the circulatory concentration of these metabolites reduces by 50–70% within 3–6 h of consumption. Several mechanisms have been reported to explain the effect of caffeine on human health. However, rarely is there a single mechanism that thoroughly explains its physiological effects. The most significant mechanism is the interaction of caffeine and adenosine. Adenosine is one of the most common vasodilators whose inhibition may cause reflex sympathetic activation. The consumption of caffeine blocks the adenosine receptors A1 and A2, influencing the autonomic nervous system (ANS) [8]. This prevents adenosine metabolism (adenosis) and consequently affects central nervous system (CNS) activity by releasing catecholamine. Also, the presence of excess adenosine stimulates the vasomotor, medullary, and respiratory networks [9]. The ANS is responsible for controlling many of the physiological functions of the body, including the vagal activities. The vagal activities include the contraction of the heart muscles, maintaining the heart rate, and the peripheral resistance of the blood vessels. The most common method that is used to detect alteration in the ANS is heart rate variability (HRV) analysis. Several studies in the literature have used HRV to find the short-term as well as the long-term effects of the consumption of caffeine and coffee on the ANS and vagal activities. In their review, Koenig et al. reported that the consumption of caffeinated beverages increases the HRV parameters [10]. Dömötör et al. have evaluated the effects of coffee on systolic blood pressure, deviation in the normal heartbeat interval, and heart rate [11]. The study showed no significant impact on any of the physiological parameters. In other research, the authors reported that the detrimental effects of coffee are only due to unhealthy habits such as smoking, alcohol use, etc. [12]. Also, adjusting these confounding factors weakens the association between coffee and vagal activities and makes the effect insignificant. Sarshin et al. have performed a dose-dependent study that found a correlation between the effect of caffeine on the autonomic cardiac activity at different caffeine dosages [13]. The authors have reported increased autonomic cardiac activity post-exercise in caffeine consumers. However, no significant effect of varying dosages of caffeine was observed. Similar results were found by Karayigit et al., showing improved muscular endurance [14]. Caffeine’s effect on the risk of cardiovascular diseases was also studied by Calderia et al. [15] and Gaeini et al. [16]. These studies reported no correlation between coffee and the risk of cardiovascular diseases. However, an increased QTc interval was reported after consuming caffeinated beverages in other research. An increased QTc interval is an indicator of arterial fibrillation [17,18]. Further, it has been reported that the consumption of warm water and decaffeinated coffee had a similar effect on the cardiac autonomic system as that of caffeinated coffee [19]. The findings of the aforesaid studies are mixed and fluctuant, making the correlation between coffee and cardiac autonomic function unclear. However, much of the literature suggests that caffeine triggers a change in HRV measures. If this is to be believed, a significant difference in cardiac functioning is also expected. These changes can be assessed by analyzing the patterns of the ECG signals.

The electrocardiogram (ECG) is the most widely used non-invasive method that tracks the changes in electro-cardio physiology and diagnoses cardiac abnormalities. Earlier studies have addressed the adverse and harmful effects of coffee in the cardiovascular system, including QRS tachycardia due to caffeine intoxication [20], an increase in the number of ventricular premature beats [21], a decrease in the right ventricular refractory period [21], and a decrease in the heart rate [22]. Though the studies mentioned above strongly suggest changes in electro-cardio physiology after consuming coffee, various authors also have reported contradicting findings [23,24]. Hence, researchers are now shifting to alternate methods to find these effects on the cardiac system. Usually, the analysis of the ECG signals follow several steps, including pre-processing, feature extraction, feature selection, and classification. The most important aspect of signal pre-processing is removing the unwanted noise from the recorded signal. The source of the noise may be due to improper electrode contact, motion artifacts, muscle contraction, baseline wander, etc. The signal gets deformed after interfering with the noise, resulting in an abnormal recording. Numerous noise removal methods that employ advanced state-of-the-art algorithms such as deep learning, convolutional neural network, adaptive discrete wavelet transform, etc., have been reported recently [25,26,27]. However, the denoising methods depend on the type of noise present within a signal. The details of the denoising method followed in this study are represented in Section 2.2. The feature that contains the most valuable and discriminative information of a signal can be obtained either from the time–domain method, frequency–domain method, joint time–frequency method, or the statistical method. However, these methods have not yet been sufficiently explored for the prospect of detecting coffee-induced effects in electro-cardio physiology. In a recent study [28], it has been reported that statistical and entropy-based features can efficiently see coffee-induced changes in an ECG. Nevertheless, these features only reflect the variations of the signals in the time domain. Due to the non-stationary nature of the ECG signal, the time–domain analysis method is sensitive to the distortion of the waveform. Further, the frequency–domain analysis method is based on the hypothesis that the input signal is stationary [29], making the application of the time–frequency analysis method foreseeable. The main advantage of time–frequency analysis is that it offers a simultaneous signal interpretation in both time and frequency.

**Figure 1:**
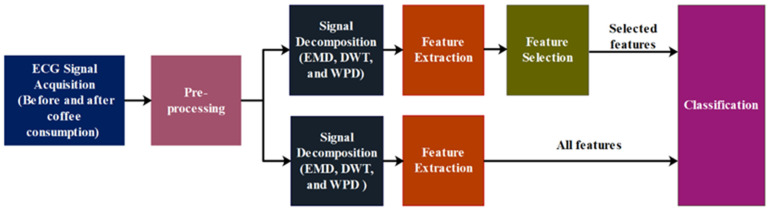
Overview of the proposed model.

Considering these facts, the current study employs time–frequency decomposition-based methods for the detection of coffee-induced effects on ECG signals. The overview of the study is represented in Figure 1. The ECG signals were recorded from the volunteers before and after their consumption of the coffee. Segments of 5 s each were extracted from the ECG signal recordings. Herein, three widely used decomposition methods, namely empirical mode decomposition (EMD), discrete wavelet transform (DWT), and wavelet packet decomposition (WPD), were employed for each 5 s ECG segment. The statistical and entropy-based features were computed from each decomposed coefficient. Wilcoxon’s signed-rank test was used to identify the features, which showed a statistically significant change after coffee consumption. Also, the changes in the extracted features at the different frequency bands were assessed using the decomposed coefficients. This was performed in order to find the range of frequencies at which the maximum change in these features was observed. Further, the two groups of data (before and after applying the stimulus) were classified using different machine learning algorithms to automatically detect the alteration in the ECG pattern. The study evaluated the performance of these machine learning algorithms along with the decomposition-based methods and proposed a machine learning model that can best detect the coffee-induced short-term effect on the ECG signals.

## 2. Materials and Methods

### 2.1. Dataset

Fifteen male volunteers aged between 18 and 26 years who were students of the National Institute of Technology Rourkela were included in the current study. All of the volunteers were leading sedentary life and had no prior history of smoking or alcohol addiction. The inclusion of these selection criteria reduces the effect of lifestyle and addictive behavior on the participants’ autonomic nervous system activities. Before recording the EGC signals, written permission was taken from the Institute Ethical Committee (Letter no: Ref.# NITRKL/IEC/FORM-2/002; dated 16 August 2017). The details of the experimental procedure were verbally explained to all of the participants. Thereafter, the volunteers who were willing to participate in the study were asked to sign a consent form in order to record their agreement.

### 2.2. Pre-Processing and Noise Elimination

The participants were informed about the place and timing of the recording in advance. They were requested to abstain from food for 2 h prior to the recording. On reaching the recording station on the day of recording, they were asked to sit in a wooden chair in a relaxed position. The ECG signals were recorded in Lead-I configuration for 5 min (Category NS) using the Vernier EKG sensor (Vernier Software & Technology Pvt. Ltd., Beaverton, OR, USA). After acquiring the pre-stimulus signal, the volunteers were served a hot cup of coffee (100 mL, 24.89 mg caffeine). After 10 min, the ECG signals were re-recorded for another 5 min (Category S).

During the acquisition of the ECG signal, the sampling frequency was maintained at 1000 Hz. A series of low-pass and high-pass filters with cut-off frequencies of 0.01 and 120 Hz were implemented in the acquisition program in order to band-limit the acquired signal. The powerline noise was rejected by using a notch filter (cut-off frequency: 50 Hz). The remaining noises (wideband noise) present in the signals were eliminated using the wavelet denoising technique. For this purpose, the DWT-based denoising method was employed. The literature has reported that the inverse DWT method can potentially denoise a signal [30]. Denoising can be done through signal reconstruction by choosing a set of details and approximation coefficients [31]. The ECG signals were decomposed using the DWT method (db6, level-8) and reconstructed using the detailed coefficients (D5–D8). The denoised ECG signals were then downsampled to 360 Hz. From the recorded 5 min signals, ECG segments of 5 s duration were extracted for further processing. A total of 630 segments were extracted from each group (pre-and post-coffee consumption), which were subsequently used for further analysis.

### 2.3. ECG Decomposition Methods

#### 2.3.1. Empirical Mode Decomposition

Empirical mode decomposition (EMD) is an iterative method that splits a signal into different frequency bands (intrinsic mode function, IMF [32]) and a residue, which corresponds to the trend of the signal. The method can extract instantaneous frequency information from nonlinear, non-stationary signals. It engages several computational stages that start with computing the upper and lower envelopes of a time-series signal (x(t)) using cubic spline interpolation. The mean (m) of the lower and upper envelopes are then subtracted from the original signal to obtain the first component (h(t) = x(t) − m). The signal component, h(t), is regarded as an IMF if the upper and lower envelopes of h(t) are symmetric and the number of zero-crossings as well as the number of extrema are equal or differ at most by one [33]. Once the signal component is identified as an IMF, it is subtracted from the original signal in order to get the residue. The next IMF is calculated by following the above steps with the residue as the input signal. This process continues until the final residue becomes constant, or the extraction of any additional IMF is not possible.

#### 2.3.2. Discrete Wavelet Transform

**Figure 2:**
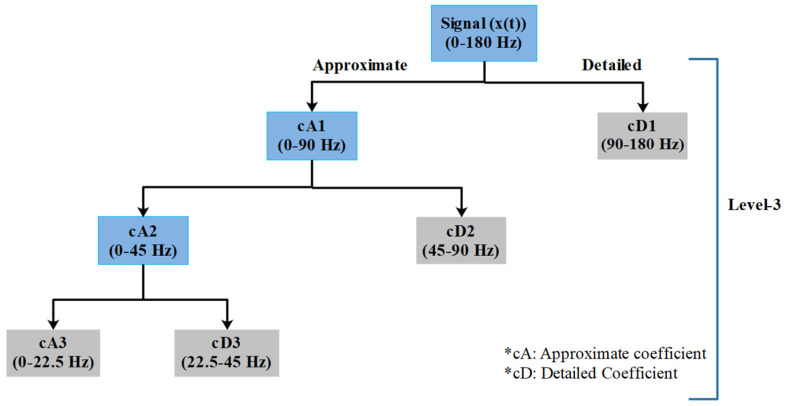
A schematic representation of the nodes from a 3-level DWT decomposition.

In signal processing, discrete wavelet transform is a widely used feature extraction technique [27]. The main idea behind this decomposition method is to divide an original signal into different resolutions by using low pass and high pass filters. The time–frequency resolution of the original waveform can be achieved using a mother wavelet ‘*Ψ*(*t*)’. In the first level of decomposition, the signal is decomposed into approximate (the low pass-filtered signal component) and detailed (the high pass-filtered signal component) coefficients [34]. Thereafter, the approximate coefficients are decomposed into the approximate and detailed coefficients (Figure 2). There are two ways of decomposing the original signal; i.e., either using an undecimated or a decimated method. Herein, the decimated DWT was employed, where the time resolution of the decomposed signal is halved. Consequently, the frequency resolution of the decomposed signal is doubled from the value of the previous level. The number of nodes that are obtained after an ‘n’ level of decomposition is n + 1. The absolute function that is used to obtain the time–frequency resolution using the mother wavelet is represented in Equation (1):

(1) $$\Psi \left(t\right)=\frac{1}{\sqrt{s}}\Psi \left(t-u\right)/s$$

where *Ψ*(*t*) is the mother wavelet, *s* is scale, and *u* is the translation parameter.

#### 2.3.3. Wavelet Packet Decomposition

**Figure 3:**
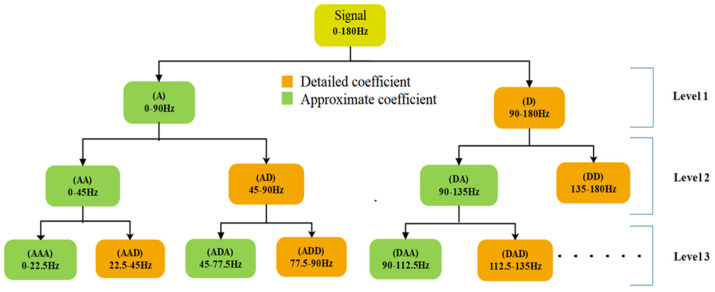
Different nodes of the WPD tree (level-3) with their frequency range.

Wavelet packet decomposition is an extension of DWT [35]. Unlike DWT, where the decompositions only occur in the approximate coefficients, WPD decomposes both the approximate and detailed coefficients (Figure 3). Since WPD can be represented as a continuous-time wavelet decomposition, sampled at the various frequency in each decomposition level, it shows better frequency resolution than DWT. A WPD of level n produces 2^n^ wavelet coefficients, which is much higher when compared to the outcome of DWT, where only *n* + 1 number of coefficients are generated. Similar to DWT, herein, the decimated WPD was followed. The wavelet decomposition function for a given decomposition level ‘*n*’ and time ‘*t_n_*’ is represented by Equation (2).

(2) $$d_{n}t_{n}=x\left(t\right)\Psi_{n}\left(\frac{t-t_{n}}{2^{n}}\right)$$

where *x*(*t*) is the input signal and *Ψ_n_* is the decomposition filter at level *n*.

### 2.4. Feature Extraction and Selection

**Figure 4:**
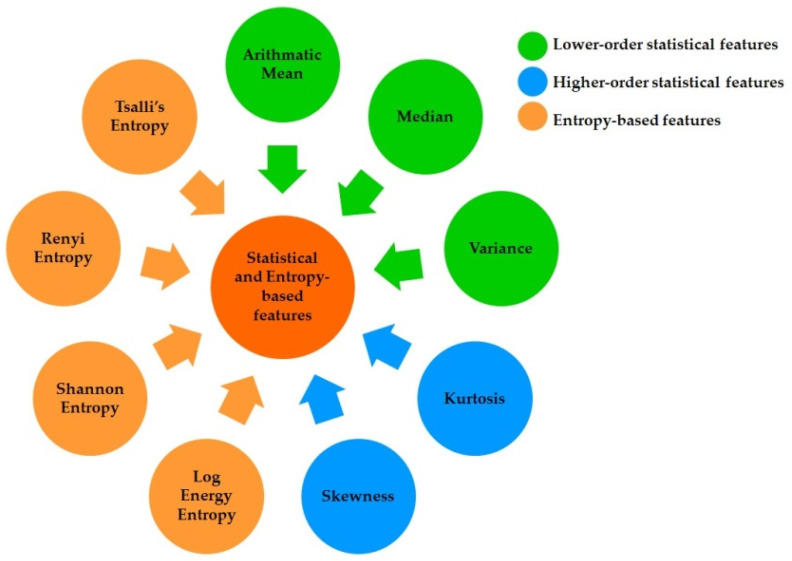
List of features used in the current study.

Feature extraction is an essential and vital step in any classification-based study [36]. It is used to extract the most informative, non-redundant values from a signal that will be used as input to the classification algorithm. Three types of features, namely lower-order statistical (LOS) features (mean, variance, and median) [36], higher-order statistical (HOS) features (kurtosis and skewness) [37], and entropy-based features (Shannon entropy, log energy entropy, Tsallis entropy, and Rényi entropy) [38], were extracted from each coefficient (DWT and WPD) and IMF (EMD) of the 5 s ECG signals. In other words, nine features (Figure 4) were computed from each decomposed signal and IMF. A statistical analysis method called Wilcoxon’s signed-rank test (WSR) was followed to obtain the significant features from the pool of features. The WSR test is a nonparametric statistical testing method that is purely designed for two-paired datasets (before and after) where the features show a non-normal behavior. The normality of the features was tested using the Shapiro–Wilk test [39]. A feature is considered statistically significant in the WSR test when the *p*-value is less than 0.05.

### 2.5. Classification Using Machine Learning Models

Machine learning is a technology that is used to develop computer algorithms that emulate human intelligence. It can deal with complex problems where conventional methods (e.g., statistical, observational, computational, etc.) are ineffective. Thus, it has been effectively used in diverse fields such as computer vision, disease detection, time-series data analysis, object and image classification, pattern recognition, etc. The present study employed nine different machine learning (ML) models, namely the generalized logistic model (GLM), linear regression (LR), decision tree (DT), naïve Bayes (NB), random forest (RF), gradient-boosted tree (GBT), support vector machine (SVM), fast large margin (FLM), and deep learning (DL). A brief description of these ML models can be found in [38,40]. Herein, the employed DL model was based on a multi-layer feed-forward neural network. The model was trained using stochastic gradient descent, which uses a back-propagation algorithm. These models were developed using RapidMiner software (Educational Version 9.5, RapidMiner Inc., Boston, MA, USA). The auto model provides a visual environment for developing automated classification and prediction models. Automatic feature engineering (AFE) was also applied during the model design. The AFE is a robust utility that helps to internally select a subset of features that are best suitable for the model performance. This can be achieved by creating hundreds of ML models and comparing the model performance with each possible feature combination [41]. Finally, the model with optimal features was considered. AFE helps in enhancing the performance of an ML model.

### 2.6. Validation and Evaluation of the ML Models

The performance of the ML models was evaluated using various matrices, namely accuracy, sensitivity, specificity, precision, and F-measure [42]. It is customary to validate the performance of the trained ML model with an independent set of data, also known as the validation set. The auto model uses a “multiset holdout” validation method that splits the input data in a 60:40 ratio. Here, the first part was used for training, and the second part was used for testing. The test data was further divided into seven subsets, and the performance of the developed model was computed using each subgroup. Then, the final performance was assessed as the average of all cases.

## 3. Results

### 3.1. EMD Analysis

**Figure 5:**
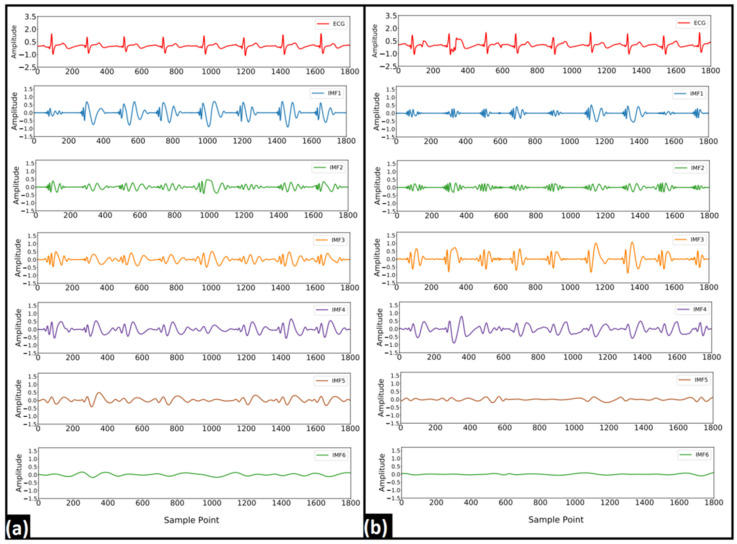
Typical IMFs of a 5 s ECG signal (**a**) before and (**b**) after consumption of coffee.

**Figure 6:**
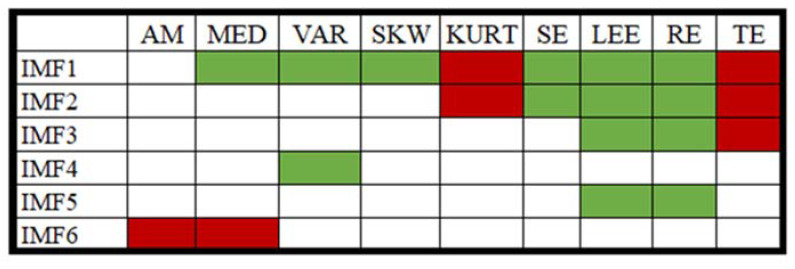
List of features that showed a significant change in different IMFs (note: 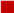: a significant decrease in value post-consumption of coffee, 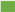: a significant increase in value post-consumption of coffee, no color: insignificant change in value post-consumption of coffee).

In EMD, six IMFs were computed from each of the ECG segments. Figure 5 represents the typical IMFs of two 5 s ECG signals recorded before and after the consumption of coffee. Several statistical and entropy-based features were computed from each of the IMFs. Since most of the extracted features showed a non-normal behavior in the Shapiro–Wilk test [39] a nonparametric testing method (WSR) was used to test the statistical significance of these features. The list of significant features (*p*-value < 0.05) obtained from each IMF is represented in Figure 6. Finally, these features were used as the input for the classification models. While evaluating the performance of the ML models, the number of IMFs varied from one to six and the ML model that showed the optimum performance (in terms of accuracy) in each case is tabulated in Table 1. Other performance indices, namely the AUC, precision, F-measure, sensitivity, and specificity of the most accurate models, are also listed in Table 1. It was observed that the DL model showed a maximum accuracy of 57.50% when the number of IMFs was 4. The details of all of the ML models that were developed have been given in Supplementary Material Table S1.

### 3.2. DWT Analysis

**Figure 7:**
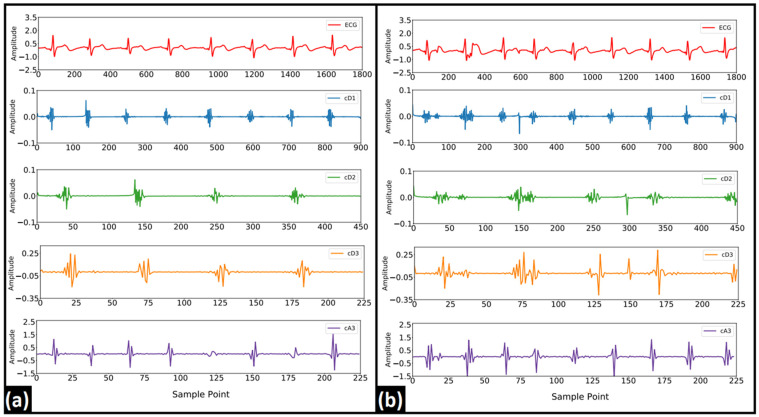
Typical DWT coefficients after level-3 decomposition using db6 mother wavelet (**a**) before and (**b**) after the consumption of coffee.

**Figure 8:**
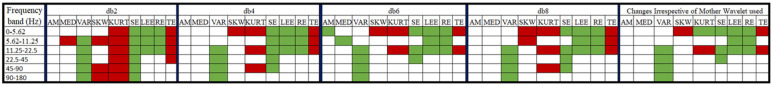
List of significant features in each frequency band (or coefficients) after a level-5 decomposition in the case of DWT (note: 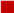: a significant decrease in value post-consumption of coffee, 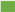: a significant increase in value post-consumption of coffee, no color: insignificant change in value).

**Figure 9:**
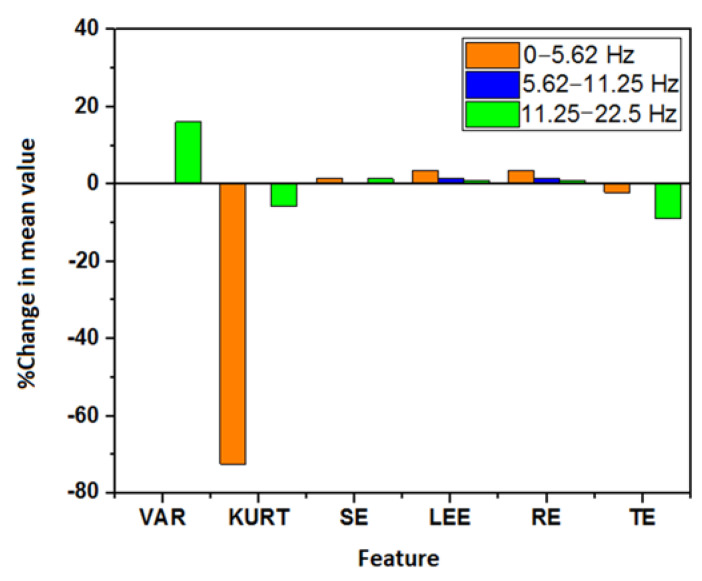
Percentage change in the mean values of the significant features obtained in the different frequency bands of the ECG signal after DWT decomposition.

The DWT-based decomposition was performed on each of the 5 s ECG signals using a range of decomposition levels (levels: 2–5) and Daubechies mother wavelets (db2, db4, db6, and db8). A representation of the obtained decomposed signals after level-4 decomposition using db4 is represented in Figure 7. Nine different features were extracted from each of the decomposed signals. Due to the non-normal distribution of many of the features in the Shapiro–Wilk test, the WSR test was employed to test the statistical significance of each of the features. In DWT, each coefficient belongs to a certain frequency band [43]. The statistically significant features that were obtained from each of the decomposed signals/coefficients at the level-5 decomposition (with each Daubechies wavelet) are represented in Figure 8. It was observed that most of the significant changes are seen in the lower frequency bands (<22.5 Hz), irrespective of the mother wavelet used. Figure 9 represents the percentage change in the mean values of the significant features in the lower frequency bands (<22.5 Hz). It is evident from the figures that the entropy value (except that of TE) has increased in the frequency range 0–5.62 Hz and 11.25–22.5 Hz. Besides this, a decrease in the kurtosis and an increase in the variance were observed in the frequency range of 0–5.62 Hz and 11.25–22.5 Hz, respectively. However, only a rise in entropy (LEE and RE) was seen in the frequency range of 5.62–11.25 Hz. The statistically important features that hold the discriminative information for the ECG signals pre- and post-stimulus conditions (coffee consumption) were then applied as input for the ML models. Table 2 illustrates the ML models that showed the best performance in each case after varying the level of decomposition and the mother wavelet. The highest performance accuracy of 78.33% ± 0.76% was achieved by the GBT model when the db6 mother wavelet was employed at the decomposition level of 2. The details of each ML model developed using the DWT method are provided in Supplementary Materials Tables S2–S5. The results suggested that the highest accuracy (78.33% ± 0.76%) was obtained in the GBT model while using the db6 mother wavelet at level-2 decomposition.

### 3.3. WPD Analysis

**Figure 10:**
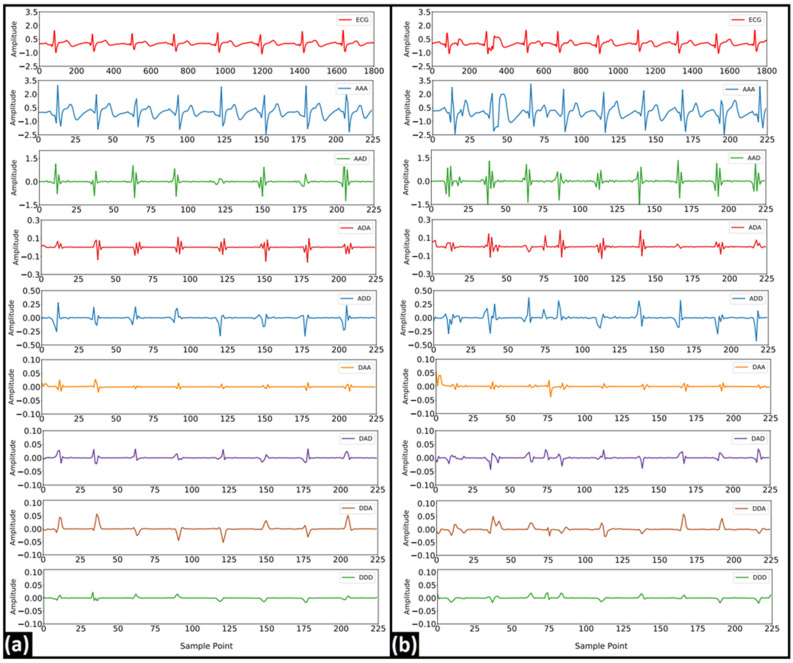
Typical WPD coefficients after level-3 decomposition using db6 mother wavelet (**a**) before and (**b**) after consumption of coffee.

**Figure 11:**
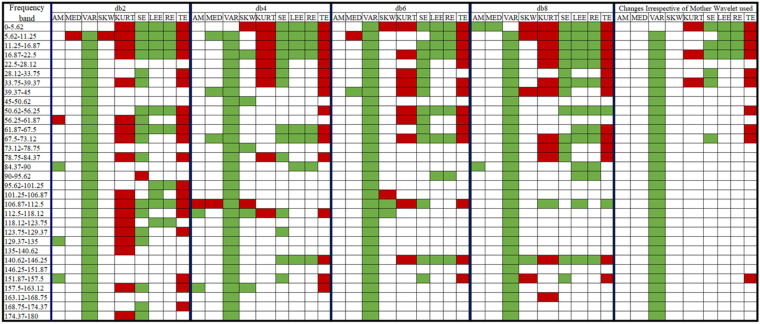
List of significant features in each frequency band (or coefficients) after a level-5 decomposition in the case of WPD (note: 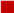: a significant decrease in value post-consumption of coffee, 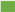: a significant increase in value post-consumption of coffee, no color: insignificant change in value post-consumption of coffee).

**Figure 12:**
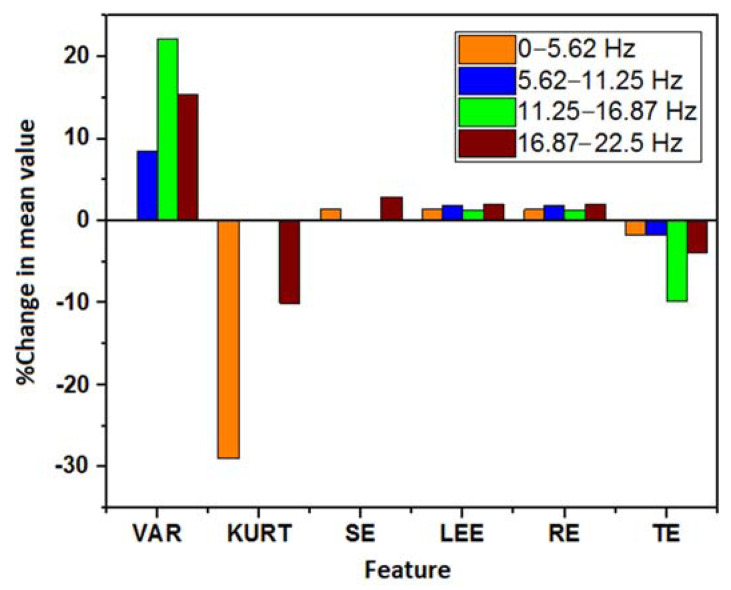
Percentage change in the mean value of the significant feature in different frequency bands.

The WPD-based decomposition was employed for each of the 5 s ECG signals. For this purpose, the level of decomposition and Daubechies mother wavelets were varied in a similar way as was described in the case of DWT. Figure 10 shows a typical representation of the decomposed ECG signals after level-3 decomposition using the db6 mother wavelet. Many of the features that were extracted from the WPD coefficient showed a non-normal distribution in the Shapiro–Wilk test. Similar to DWT, the coefficients of WPD contain a certain frequency band that depends on the sampling frequency and the level of decomposition (Figure 3). A representation of the significant features that were obtained from each frequency band at the level-5 decomposition is given in Figure 11. It was observed that most of the changes in the ECG signal after the consumption of coffee were confined to the lower frequency band (<22.5 Hz). Figure 12 represents the percentage change in the mean value of the significant features from the aforesaid lower frequency band. This was irrespective of the type of mother wavelet that was used during the decomposition process. It is evident from Figure 12 that a rise in the variance value was observed in the frequency band 5.62–22.5 Hz. Most of the entropy values (except TE) also showed a hike after the consumption of coffee. Moreover, a decrease in kurtosis was also observed in the frequency ranges of 5.62–11.25 Hz and 16.87–22.5 Hz.

For a decomposition level “L”, the features that hold discerning information for the ECG signal before and after applying the stimulus (coffee) were used as an input into the ML models. Table 3 illustrates the models that showed the best accuracy when the decomposition level and type of Daubechies wavelet were varied. The GBT model developed using the db2 mother wavelet and a decomposition level of 4 showed the highest performance accuracy of 73.33% ± 1.16%. The details of each ML model created using the WPD-method are provided in Supplementary Materials Tables S6–S9.

We have also analyzed the performance of the ML models using all of the features irrespective of the IMFs, decomposition level, and mother wavelets. This was done in order to validate the efficiency of the proposed feature selection method. Table 4 represents the performance of the best two ML models in terms of accuracy. The details of all of the other model performances are given in Supplementary Material Table S10.

## 4. Discussions

In this study, three joint time–frequency decomposition methods have been compared for the detection of short-term coffee-induced changes in ECG patterns. ECG segments of 5 s duration from the participants in the pre- and post-stimulus conditions were obtained. This segment-based analysis is a well-documented method. ECG segments of different lengths have been used in various studies [44,45,46]. Though there is no clinical evidence that is in support of choosing a duration of 5 s, Sinha et al. compared the performance of the classification model at different signal lengths. They found optimum results when the signals of 5 s duration were used [47]. Thus, a segment of 5 s duration was chosen in this study. The ECG segments were decomposed using the EMD, DWT, and WPD methods. The features that were extracted from the decomposed coefficients were then classified using several ML models (e.g., GLM, LR, DT, NB, RF, GBT, SVM, FLM, and DL). The performance evaluation is represented in Table 1, Table 2 and Table 3. The decomposition parameters were varied in order to find out the most suitable number of IMFs in EMD and the decomposition levels in the case of DWT and WPD. It is common to choose a mother wavelet that shows structural similarity with the original signal [48]. Herein, different mother wavelets from the Daubechies family (db2, db4, db6, and db8) were chosen for the analysis. This is due to the structural similarity that they possess with the QRS-complex of the ECG signal [49,50]. Though the coefficients that were obtained after the decomposition process reveal the characteristics of the ECG signal, for a better interpretation it is inevitable to find various features that hold the valuable and discriminative information between the two groups of data [38].

Various feature extraction methods have been reported in the literature for detecting the effects of coffee on cardiac physiology (Table 5). However, the most studied features are the ECG-based morphological features. This includes the amplitude metrics (e.g., P-wave, T-wave, R-peak, etc.) and wave intervals (e.g., RR interval, QTc interval, etc.). Uddin et al. have used the amplitude of R-peak, P-wave, and T-wave to evaluate the changes in ECGs after caffeine consumption [51]. The authors observed no significant increase in the values of R-peak. However, the amplitude of the P- and T-wave had decreased. In another research paper, QTc interval was used to evaluate the changes in the electrocardiography and detect whether caffeine results in cardiac arrhythmias [52,53]. This is because the QTc prolongation is an indicator of increased cardiac risk. However, they found no significant change in the QTc interval. The results that were reported in [17,54,55] contradicted those aforesaid findings with a substantial increase in the QTc after consumption of the caffeinated beverage. Table 5 compares the findings of various studies that have used morphological features in order to evaluate the cardiac change after coffee consumption. The comparison among these studies infers that the correlation between caffeine/coffee consumption and an alteration in electro-cardio physiology is not equivocal. The reason may be that the morphological features are not efficient in tracking the minute changes in the ECG patterns. In our previous study [28], it has been reported that the statistical and entropy-based features that are obtained from 5 s ECG segment can efficiently detect the variation in ECG patterns due to coffee-induced short-term effects. These features reflect only the changes in the time domain. No attempt was made to study the variation in the frequency domain’s information within ECG signals. Hence, herein these said feature extraction methods and the joint time–frequency decomposition methods were employed. The lower order statistical features (such as mean and variance) are of lower complexities and are independent of the fiducial points of the ECG signals. The higher-order statistical features, such as skewness and kurtosis, are related to the signal’s shape and contain amplitude and phase information [28]. In an ECG signal, the skewness measures the symmetry of the signal around the R-peak, whereas the kurtosis represents whether the distribution of a signal is heavy or light-tailed compared to the normal distribution [56]. The inclusion of entropy-based features provides information regarding the rate of generation of the information in a dynamic system. It can be used as a measure of complexity in biomedical signal analysis [21,57]. Entropy-based features have also been employed in [58] to measure the changes in the nonlinear HRV measures after caffeine ingestion. This study observed an increase in the variance and entropy (except TE) values in both wavelet-based methods (Figure 9 and Figure 12). Likewise, in the case of EMD, the entropy values (except TE) were significantly higher in the first two IMFs. Interestingly, in the higher IMFs only a few features have shown significant change. This reflects an increase in the irregularity of the ECG patterns post-coffee consumption. Moreover, a higher level of entropy also reflects an increase in the degree of uncertainty in the ECG patterns after the consumption of coffee.

In other research [12,58,59], the changes in the frequency–domain parameters have been well evaluated in HRV analyses to detect alterations in the autonomic nervous system (ANS) after caffeine intake. However, the variation in the statistical measures in different frequency bands has not yet been satisfactorily explored. It has also been reported that the most useful information in an ECG signal lies within the frequency range of 0–30 Hz [61]. The highest amplitude of a normal P and T wave occurs at 3 Hz, whereas for the QRS complex, it is 15 Hz. Hence, it is quite expected that the variation in the ECG patterns will be more pronounced in this frequency range. We found similar results when the variations in the said features (those which were statistically significant) were compared in the different frequency bands. Our results (Figure 8 and Figure 11) suggest most of these changes are localized in the lower frequency bands (<22.5 Hz) irrespective of the type of mother wavelet that was used. The EMD method was overlooked for the frequency band analysis as the frequency range of the IMFs is non-uniform [62].

The current study also proposes an ML model for detecting alteration in the ECG signal pattern due to coffee-induced short-term effects. Several state-of-the-art ML models have been compared in order to find the best classifier that can efficiently detect the alterations in cardiac electrophysiology. The study proposes that the GBT classifiers, when employed to the features obtained after DWT decomposition (using db6 mother wavelet and level-2 decomposition), show the best performance (Table 2). In Table 5 the proposed method was compared with other reported literature.

In the research of Pradhan and Pal [28], the performance of different tree-based ML models was tested in order to detect coffee-induced short-term effects. The results suggested that random forest was the best ML model with mean accuracy, sensitivity, and AUC of 75.1%, 75.0%, and 84.4%, respectively. The GBT model, as proposed in the current study, shows a mean accuracy, sensitivity, and AUC of 78.33%, 83.3%, and 86.6%, respectively. In order to validate the efficiency of the feature selection method that was used in the current study, we have also assessed the performance of these models when all of the extracted features were used as inputs. In the case of DWT and WPD, the GBT showed better performance with the proposed feature selection method. Interestingly, the DL showed an increased performance with the larger feature set compared to when the feature selection method was employed. This result was expected as the deep learning model performs comparatively better with larger datasets. However, the performance of the DL models was lower than that of the GBT model. Lastly, when the EMD-based decomposition method was used, the ML models performed poorly compared to the performance of the wavelet-based methods.

## 5. Limitations, Future Work, and Conclusions

This study shows a new way to detect alterations in ECG signals due to coffee-induced short-term physiological effects. To the best of our knowledge, this is the first study that compares various joint time–frequency decomposition methods (EMD, DWT, and WPD) in order to investigate the influence of coffee on electro-cardio physiology. The results obtained in the statistical analysis confirm that many features possess statistically discriminative information in different frequency bands of the ECG signals in the pre-and post-stimulus conditions. Also, it was observed that most of these changes are localized in the lower frequency band (<22.5 Hz), where the signal contains the majority of the information. This confirms the alteration in cardiac activity after the consumption of coffee. However, these results are unable to express the exact change in the ECG morphological features. Also, the study suggested a range of frequencies in which most of the changes in the statistical parameters were localized. However, information about the exact frequency value is lacking. It is worth noting that our experiment was performed in a controlled environment. Hence, changes in the environmental conditions, physical status, habitual information, and caffeine/coffee dosage may produce different results. Hence, the effects of these parameters on electro-cardio physiology and the model performance will be explored in the future. A potential extension to this study would be to design an ML model that can detect the influence of coffee and predict the dosage by observing the changes in the ECG signal patterns. Caffeine possesses an ergogenic effect and is found to improve performance in many sports, including rifle shooting [63], swimming [64], handball [65], weightlifting [66], etc. However, the World Anti-Doping Agency (WADA) has not included caffeine in its prohibited list but rather in its monitoring program [67]. This study, in the future, maybe employed to find the caffeine dosage in athletes by using their ECG signals. Numerous other advanced state-of-the-art decomposition methods such as variable mode decomposition [68], flexible analytic wavelet transform [58], and classification methods will be explored in the future. A limitation of the current study can be the large feature set as it is associated with longer training time and large memory. Hence, as future work, it will be interesting to see how advanced deep learning algorithms such as recurrent neural networks and convolutional neural networks can be employed in the current data set and how they impact the classification’s performance. The findings of this study will open up new possibilities for drug and alcohol detection in the human body with the help of ECG signals. The current research can also be employed in the future to recognize the changes in cardiac activities after the consumption of other caffeinated beverages (e.g., tea, cola, soft drinks, etc.), drugs, and alcohol.

## Figures and Tables

**Table 1:** Classification performance of the best ML models generated from the EMD-based processing of the ECG signals at different decomposition levels.

No. of IMFs	ML Model	Accuracy	Precision	F-Measure	Sensitivity	Specificity	AUC
1	DL	56.11 ± 2.11	54.08 ± 1.59	65.21 ± 0.89	82.22 ± 2.48	30.00 ± 6.02	0.615 ± 0.055
2	GBT	53.61 ± 2.52	52.77 ± 1.91	59.25 ± 3.64	67.78 ± 7.24	39.44 ± 5.34	0.579 ± 0.019
3	FLM	53.89 ± 2.67	53.10 ± 2.26	59.69 ± 2.02	68.33 ± 4.21	39.44 ± 6.63	0.551 ± 0.041
4	DL	57.50 ± 2.32	57.12 ± 2.08	58.48 ± 3.01	60.00 ± 4.65	55.00 ± 3.04	0.587 ± 0.047
5	DL	53.06 ± 2.06	52.10 ± 1.39	61.84 ± 1.75	76.11 ± 3.17	30.00 ± 3.62	0.565 ± 0.050
6	GBT	53.61 ± 1.24	52.73 ± 1.04	60.66 ± 1.17	71.67 ± 5.34	35.56 ± 7.71	0.554 ± 0.024

NB: Color scale used in the table (column-wise): minimum value
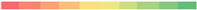
maximum value.

**Table 2:** Classification performance of the best ML models generated from the DWT-based processing of the ECG signals at different decomposition levels.

Level	Wavelet Used	ML Model	Accuracy	Precision	F-Measure	Sensitivity	Specificity	AUC
2	db2	GBT	75.28 ± 0.62	88.55 ± 3.90	70.20 ± 1.31	58.33 ± 3.40	92.22 ± 3.62	0.830 ± 0.020
	db4	GBT	70.00 ± 2.11	67.00 ± 2.98	72.59 ± 1.50	79.44 ± 4.21	60.58 ± 6.63	0.794 ± 0.009
	db6	GBT	78.33 ± 0.76	75.89 ± 2.01	79.31 ± 1.53	83.33 ± 5.20	73.33 ± 4.21	0.866 ± 0.029
	db8	GBT	73.61 ± 0.98	74.61 ± 3.04	73.11 ± 2.55	72.22 ± 7.08	75.00 ± 6.21	0.807 ± 0.017
3	db2	GBT	72.50 ± 3.73	77.44 ± 2.19	69.54 ± 5.29	63.33 ± 7.71	81.67 ± 1.52	0.810 ± 0.048
	db4	GBT	76.94 ± 3.20	79.91 ± 4.60	75.83 ± 3.04	72.22 ± 2.78	81.67 ± 5.05	0.850 ± 0.041
	db6	GBT	75.83 ± 3.49	75.18 ± 5.09	76.33 ± 2.90	77.78 ± 3.93	73.89 ± 7.24	0.839 ± 0.033
	db8	GBT	73.06 ± 2.11	87.62 ± 3.76	66.56 ± 3.62	53.89 ± 5.05	92.22 ± 3.04	0.817 ± 0.030
4	db2	GBT	72.50 ± 3.73	77.44 ± 2.19	69.54 ± 5.29	63.33 ± 7.71	81.67 ± 1.52	0.810 ± 0.048
	db4	GBT	72.50 ± 3.85	82.28 ± 6.84	67.71 ± 4.67	57.78 ± 5.34	87.22 ± 6.09	0.817 ± 0.053
	db6	GBT	69.72 ± 3.85	72.54 ± 4.77	67.65 ± 5.33	63.89 ± 8.56	75.56 ± 6.33	0.778 ± 0.034
	db8	DL	64.44 ± 3.34	64.38 ± 3.66	64.62 ± 3.35	65.00 ± 4.65	63.89 ± 5.20	0.695 ± 0.051
5	db2	GBT	70.00 ± 1.58	71.65 ± 3.40	68.96 ± 1.24	66.67 ± 3.40	73.33 ± 5.41	0.771 ± 0.032
	db4	GBT	71.11 ± 0.62	74.18 ± 2.16	69.21 ± 1.05	65.00 ± 3.17	77.22 ± 3.62	0.773 ± 0.023
	db6	GBT	68.89 ± 3.04	69.09 ± 2.53	68.60 ± 4.09	68.33 ± 6.69	69.44 ± 3.40	0.776 ± 0.041
	db8	GBT	66.67 ± 2.41	68.15 ± 3.99	65.42 ± 3.16	63.33 ± 6.33	70.00 ± 6.63	0.752 ± 0.023

NB: Color scale used in the table (column-wise): minimum value
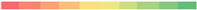
maximum value.

**Table 3:** Classification performance of the best ML models generated from the WPD-based processing of the ECG signals at different decomposition levels.

Level	Wavelet Used	ML Model	Accuracy	Precision	F-Measure	Sensitivity	Specificity	AUC
2	db2	GBT	71.11 ± 4.21	69.37 ± 3.76	72.22 ± 4.12	75.56 ± 4.56	66.67 ± 3.93	0.794 ± 0.053
	db4	GBT	69.17 ± 2.67	74.92 ± 3.85	65.15 ± 3.58	57.78 ± 4.56	80.56 ± 3.93	0.774 ± 0.032
	db6	GBT	67.78 ± 5.93	70.59 ± 7.51	65.45 ± 6.62	61.11 ± 6.51	74.44 ± 6.63	0.715 ± 0.064
	db8	GBT	71.67 ± 1.58	67.12 ± 1.35	74.99 ± 1.59	85.00 ± 3.17	58.33 ± 2.78	0.828 ± 0.025
3	db2	GBT	69.44 ± 1.96	88.39 ± 4.35	59.43 ± 4.02	45.00 ± 4.97	93.89 ± 3.04	0.787 ± 0.033
	db4	GBT	67.22 ± 2.88	84.19 ± 7.31	56.51 ± 4.69	42.78 ± 5.05	91.67 ± 4.38	0.769 ± 0.048
	db6	GBT	69.44 ± 3.80	70.82 ± 5.09	68.63 ± 3.10	66.67 ± 1.96	72.22 ± 6.51	0.786 ± 0.050
	db8	GBT	66.94 ± 4.75	65.92 ± 4.67	68.07 ± 4.55	70.56 ± 6.09	63.33 ± 6.63	0.733 ± 0.047
4	db2	GBT	73.33 ± 1.16	73.13 ± 3.02	73.50 ± 2.50	74.44 ± 7.45	72.27 ± 6.51	0.795 ± 0.046
	db4	GBT	66.11 ± 4.24	65.77 ± 4.01	66.39 ± 4.78	67.22 ± 6.92	65.00 ± 5.05	0.730 ± 0.027
	db6	GBT	68.61 ± 3.75	76.72 ± 7.07	63.25 ± 3.69	53.89 ± 2.48	83.33 ± 5.89	0.724 ± 0.052
	db8	GBT	62.22 ± 2.28	59.47 ± 1.99	67.15 ± 1.65	77.22 ± 3.04	47.22 ± 5.20	0.685 ± 0.033
5	db2	GBT	63.61 ± 2.48	67.50 ± 4.60	59.41 ± 2.53	53.33 ± 4.12	73.89 ± 6.39	0.698 ± 0.032
	db4	DL	64.17 ± 3.85	64.12 ± 4.01	64.27 ± 3.74	64.44 ± 3.62	63.89 ± 4.39	0.679 ± 0.050
	db6	GBT	61.39 ± 4.33	70.25 ± 9.33	51.28 ± 4.35	40.56 ± 3.17	82.22 ± 7.24	0.696 ± 0.064
	db8	GBT	63.06 ± 3.34	66.29 ± 2.54	58.48 ± 6.44	52.78 ± 9.42	73.33 ± 3.73	0.650 ± 0.054

NB: Color scale used in the table (column-wise): minimum value
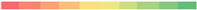
maximum value.

**Table 4:** Classification performance of the best two ML models after feeding all extracted features in each decomposition method individually and simultaneously.

Decomposition Method Used	ML Model	Accuracy	Precision	Recall	F-Measure	Sensitivity	Specificity	AUC
EMD	DL	56.9 ± 2.2	57.2 ± 2.9	56.7 ± 4.6	56.8 ± 2.2	56.7 ± 4.6	57.2 ± 6.7	0.576 ± 0.038
	GLM	53.6 ± 2.1	52.8 ± 1.5	67.8 ± 5.0	59.3 ± 2.7	67.8 ± 5.0	39.4 ± 3.6	0.573 ± 0.021
DWT	GBT	76.9 ± 2.7	84.5 ± 2.6	66.1 ± 6.0	74.0 ± 3.8	66.1 ± 6.0	87.8 ± 2.5	0.859 ± 0.041
	DL	70.6 ± 2.7	69.6 ± 3.4	73.3 ± 1.5	71.4 ± 2.0	73.3 ± 1.5	67.8 ± 5.0	0.800 ± 0.035
WPD	DL	68.3 ± 6.1	69.5 ± 6.9	65.6 ± 5.4	67.5 ± 6.1	65.6 ± 5.4	71.1 ± 7.0	0.759 ± 0.049
	GBT	66.9 ± 3.6	68.7 ± 3.9	62.8 ± 9.3	65.3 ± 5.2	62.8 ± 9.3	71.1 ± 6.4	0.767 ± 0.043
EMD + DWT + WPD	GBT	69.7 ± 4.9	66.3 ± 3.9	80.0 ± 6.6	72.5 ± 4.8	80.0 ± 6.6	59.4 ± 4.6	0.790 ± 0.045
	DL	68.9 ± 2.9	71.1 ± 3.5	63.9 ± 5.9	67.2 ± 3.7	63.9 ± 5.9	73.9 ± 4.6	0.775 ± 0.027

**Table 5:** Comparison of the various studies on the detection/classification of coffee/caffeine-induced changes in the cardiac autonomic and electrocardiographic parameters.

Problem	Methods	Parameters/Features	Results/Observation	Reference
Coffee/caffeine-induced changes in the cardiac autonomic function	HRV analysis	Time–domain parameters: RMSSD, SDNN, pNN50, mean RRI Frequency domain parameters: HF, LF	A reduced trend in the HRV vagal indexes was observed for people who consumed ≥3 cups of coffee/day	[12]
	HRV analysis	Vagal parameters: heart rate, blood pressure Time–domain parameters: pNN50, RMSSD Frequency domain parameters: HF, LF. VLF, LF/HF	Lower HR, higher blood pressure, a significant rise in HF power, significant rise in time–domain parameters	[59]
	HRV analysis	Nonlinear parameters: correlation dimension, approximate entropy, detrend fluctuation parameters	Coffee and cola showed no significant effect on the nonlinear parameter of the HRV	[58]
	ECG morphology-based statistical analysis	Electrocardiographic parameters: R-peak, P-wave, and T-wave	No significant increase in the amplitude of R-peak, decrease in the value of P- and T-peaks	[51]
	ECG morphology-based statistical analysis	Vagal parameters: heart rate, blood pressure. Electrocardiographic parameters: RR interval, QTc interval	No changes in the diastolic blood pressure, decrease in the heart rate, no change in QTc interval	[52]
	ECG morphology-based statistical analysis	Electrocardiographic parameters: mean RR interval, QTc interval	No significant prolongation in the QTc interval, a significant decrease in the heart rate	[53]
	ECG morphology-based statistical analysis	Vagal parameters: blood pressure, heart rate. Electrocardiographic parameters: PR interval, QRS duration, QTc interval	No significant change in any parameter after having the energy drink	[24]
	ECG morphology-based statistical analysis	Vagal Parameters and ECG morphological parameters	Increased blood pressure (systolic and diastolic) and prolonged QTc interval	[54]
	ECG morphology-based statistical analysis	Vagal parameters: blood pressure, heart rate Electrocardiographic parameters: PR interval, QRS duration, QTc interval	An increase in systolic blood pressure, no significant change in the electrocardiography parameters	[60]
	ECG morphology-based statistical analysis	Vagal parameters: systolic and diastolic blood pressure Electrocardiographic parameters: QT interval	Increased heart rate, blood pressure (systolic and diastolic), and QT interval	[55]
	ECG morphology-based statistical analysis	Vagal parameters: blood pressure, heart rate Electrocardiographic parameters: PR interval, QRS duration, QTc interval	Prolonged QTc interval and increased blood pressure (systolic and diastolic).	[17]
	Decomposition based analysis (DWT and WPD)	Statistical and entropy features	Increase in the variance and entropy-features, the changes are mostly reflected in the lower frequency range in the ECG signal (<22.5 Hz)	Proposed Study
Automatic detection of the coffee-induced changes in the ECG signals	ECG segment based statistical analysis	Statistical and entropy features	Accuracy: 75% (random forest classifier)	[28]
	ECG signal decomposition-based statistical analyses. (EMD, DWT, and WPD)	Statistical and entropy features	Accuracy: 78% (gradient-boosted tree classifier)	Proposed Study

## Data Availability

Not applicable.

## Supplementary Materials

The following supporting information can be downloaded at: https://www.mdpi.com/article/10.3390/nu14040885/s1, Table S1: Classification performance of the ML models generated from the EMD-based processing of the ECG signals, Tables S2–S5: Classification performance of the ML models generated from the DWT-based processing of the ECG signals at a decomposition level 2–5, Tables S6–S9: Classification performance of the ML models generated from the WPD-based processing of the ECG signals at a decomposition level 2–5, Table S10: Classification performance of the ML models generated after employing all extracted features in each decomposition method.
