# Supplementary material for: Automated Detection of Caffeinated Coffee-Induced Short-Term Effects on ECG Signals Using EMD, DWT, and WPD
Source: Nutrients. 2022 Feb 19;14(4):885. doi: 10.3390/nu14040885 (PMC8875083; doi:10.3390/nu14040885)
Supplement: Supplementary file 1 [file nutrients-14-00885-s001.zip › nutrients-1587886-SI.pdf]

nutrients

MDPI

Supplementary Information:

1.EMD

Table S1. Classification performance of the best ML models generated from the EMD-based processing of the ECG signals

No. of IMFs	ML-model	Accuracy	Precision	Recall	F-measure	Sensitivity	Specificity	AUC
1	DT	50.00±3.40	50.00±6.24	28.89±4.21	36.57±4.82	28.89±4.21	71.11±4.21	0.529±0.070
	LR	51.39±1.96	50.72±1.01	98.89±1.52	67.05±1.05	98.89±1.52	3.89±3.73	0.527±0.086
	GLM	50.28±1.16	50.14±0.59	98.33±1.52	66.42±0.71	98.33±1.52	2.22±2.32	0.526±0.086
	NB	50.83±3.34	50.51±1.92	88.89±2.78	64.40±2.04	88.89±2.78	12.78±5.76	0.553±0.075
	RF	52.78±1.96	76.29±15.76	8.89±4.97	15.47±8.05	8.89±4.97	96.67±2.32	0.521±0.062
	GBT	50.00±2.20	50.00±1.36	80.56±5.20	61.66±2.14	80.56±5.20	19.44±5.20	0.517±0.046
	DL	56.11±2.11	54.08±1.59	82.22±2.48	65.21±0.89	82.22±2.48	30.00±6.02	0.615±0.055
	SVM	44.44±4.91	43.38±6.19	40.00±10.13	41.50±8.24	40.00±10.13	48.89±3.17	0.425±0.056
	FLM	54.72±2.32	0.572±0.047	52.74±1.30	90.56±4.21	60.65±2.02	90.56±4.21	0.572±0.047
2	DT	46.94±3.60	48.07±2.42	71.67±5.34	57.44±2.11	71.67±5.34	22.22±10.02	0.469±0.036
	LR	53.06±5.05	52.73±4.38	59.44±5.76	55.85±4.80	59.44±5.76	46.67±6.33	0.537±0.065
	GLM	53.06±4.54	53.14±4.53	52.22±4.56	52.66±4.44	52.22±4.56	53.89±5.41	0.531±0.049
	NB	51.11±3.85	51.27±4.87	36.67±8.65	42.50±7.20	36.67±8.65	65.58±5.41	0.576±0.044
	RF	48.33±3.46	46.37±6.88	22.78±5.34	30.44±6.12	22.78±5.34	73.89±4.65	0.517±0.082
	GBT	53.61±2.52	52.77±1.91	67.78±7.24	59.25±3.64	67.78±7.24	39.44±5.34	0.579±0.019
	DL	51.11±1.16	50.67±0.70	82.78±3.62	62.85±1.54	82.78±3.62	19.44±1.96	0.550±0.028
	SVM	48.61±4.50	48.24±6.07	35.00±5.76	40.40±5.48	35.00±5.76	62.22±8.47	0.465±0.066
	FLM	51.67±1.81	57.32±7.53	13.33±2.32	21.58±3.40	13.33±2.32	90.00±2.48	0.596±0.061
3	DT	49.44±0.76	40.00±0.00	2.22±1.24	4.21±0.00	2.22±1.24	96.67±2.32	0.458±0.040
	LR	51.94±1.58	51.43±1.15	70.00±3.62	59.27±1.78	70.00±3.62	33.89±3.62	0.522±0.016
	GLM	50.00±0.98	49.80±3.58	17.22±3.62	25.46±4.43	17.22±3.62	82.78±3.04	0.526±0.063
	NB	52.78±4.05	55.02±7.43	28.89±5.76	37.78±6.62	28.89±5.76	76.67±3.17	0.561±0.046
	RF	49.72±1.16	45.45±0.00	2.78±1.96	-	2.78±1.96	96.67±3.04	0.589±0.055
	GBT	53.33±3.75	52.04±2.27	87.22±3.17	65.16±2.33	87.22±3.17	19.44±6.51	0.572±0.059
	DL	53.61±2.88	52.21±1.73	86.11±3.40	64.99±2.05	86.11±3.40	21.11±4.65	0.546±0.043
	SVM	53.06±1.52	52.08±1.11	78.33±4.97	62.49±1.36	78.33±4.97	27.78±6.80	0.554±0.065
	FLM	53.89±2.67	53.10±2.26	68.33±4.21	59.69±2.02	68.33±4.21	39.44±6.63	0.551±0.041
4	DT	50.00±1.70	50.00±0.00	3.33±2.32	6.25±0.00	3.33±2.32	96.67±3.62	0.523±0.066
	LR	51.67±2.08	51.35±1.71	65.00±5.76	57.28±2.44	65.00±5.79	38.33±6.63	0.546±0.052
	GLM	48.89±2.85	49.04±2.36	58.89±6.33	53.44±3.68	58.89±6.33	38.89±5.20	0.530±0.030
	NB	58.06±3.32	59.54±3.64	50.00±5.89	54.28±4.63	50.00±5.89	66.11±3.62	0.596±0.056
	RF	48.61±1.39	49.29±0.71	96.11±1.52	65.16±0.95	96.11±1.52	1.11±1.52	0.472±0.053
	GBT	50.56±3.20	50.16±3.44	46.11±11.88	47.67±7.93	46.11±11.88	55.00±6.92	0.529±0.039
	DL	57.50±2.32	57.12±2.08	60.00±4.65	58.48±3.01	60.00±4.65	55.00±3.04	0.587±0.047
	SVM	46.11±1.16	47.19±0.89	65.56±4.65	54.83±2.02	65.56±4.65	26.67±4.21	0.437±0.035
	FLM	50.00±0.00	48.67±36.64	3.33±3.04	6.25±0.00	3.33±3.04	96.67±2.32	0.451±0.046
5	DT	NA	-	-	-	-	-	-
	LR	51.67±3.73	51.11±2.35	82.78±3.62	63.15±2.15	82.78±3.61	20.56±8.00	0.555±0.049
	GLM	52.22±0.76	51.57±0.59	73.89±7.51	60.62±2.34	73.89±7.51	30.58±7.61	0.560±0.040

	NB	52.22±4.00	51.59±2.86	74.44±3.04	60.93±2.82	74.44±3.04	30.00±6.02	0.564±0.072
	RF	NA	-	-	-	-	-	-
	GBT	52.50±1.81	53.82±2.66	34.44±7.51	41.68±5.98	34.44±7.51	70.56±5.76	0.526±0.047
	DL	53.06±2.06	52.10±1.39	76.11±3.17	61.84±1.75	76.11±3.17	30.00±3.62	0.565±0.050
	SVM	48.33±3.73	48.22±3.90	47.78±6.63	47.92±5.03	47.78±6.63	48.89±5.05	0.491±0.043
	FLM	48.61±4.71	49.16±2.76	83.33±6.21	61.82±3.81	83.33±6.21	13.89±4.81	0.485±0.077
6	DT	49.44±2.32	49.71±1.20	97.22±3.40	65.78±1.82	97.22±3.40	1.67±1.52	0.492±0.032
	LR	52.50±3.01	52.07±2.64	60.00±7.51	55.66±4.53	60.00±7.51	45.00±4.12	0.530±0.043
	GLM	48.61±4.91	47.92±7.06	33.89±7.45	39.55±7.02	33.89±7.45	63.33±6.63	0.494±0.071
	NB	51.94±1.58	52.68±2.11	37.78±2.48	43.99±2.37	37.78±2.48	66.11±1.24	0.513±0.048
	RF	48.33±1.16	49.1±0.61	95.56±2.48	64.90±1.06	95.56±2.48	1.11±1.52	0.473±0.055
	GBT	51.94±1.86	53.58±3.47	26.11±6.39	34.87±6.70	26.11±6.39	77.78±3.40	0.538±0.32
	DL	53.61±1.24	52.73±1.04	71.76±5.34	60.66±1.17	71.67±5.34	35.56±7.71	0.554±0.024
	SVM	53.61±0.76	53.70±0.90	52.78±3.93	53.16±1.93	52.78±3.94	54.44±4.21	0.515±0.042
	FLM	48.33±3.01	30.00±32.60	2.78±3.40	5.10±0.00	2.78±3.40	93.89±3.04	0.480±0.101

2. DWT

Table S2. Classification performance of the best ML models generated from the DWT-based processing of the ECG signals at a decomposition level of 2.

Wavelet	ML-model	Level-2						
		Accuracy	Precision	Recall	F-measure	Sensitivity	Specificity	AUC
Db2	DT	56.11±1.58	69.85±10.76	23.33±3.73	34.55±3.36	23.33±3.73	88.89±5.89	0.608±0.053
	LR	65.28±3.40	80.65±4.25	40.00±5.76	53.38±5.98	40.00±5.76	90.56±1.52	0.706±0.031
	GLM	57.22±2.06	83.47±11.78	18.33±3.17	29.02±4.36	18.33±3.17	96.11±3.17	0.684±0.055
	NB	60.28±4.67	67.35±8.69	40.56±4.21	50.55±5.26	40.56±4.21	80.00±6.33	0.623±0.031
	RF	57.22±4.21	70.56±13.92	25.56±4.97	37.33±6.47	25.56±4.97	88.89±5.56	0.621±0.070
	GBT	75.28±0.62	88.55±3.90	58.33±3.40	70.20±1.31	58.33±3.40	92.22±3.62	0.830±0.020
	DL	64.72±3.34	68.01±5.18	56.11±4.97	61.36±3.82	56.11±4.97	73.33±5.76	0.719±0.010
	SVM	56.11±4.46	56.68±4.59	52.22±6.33	54.25±5.03	52.22±6.33	60.00±6.39	0.589±0.053
	FLM	54.17±3.11	52.72±2.03	81.11±3.62	63.89±2.37	81.11±3.62	27.22±4.56	0.546±0.046
Db4	DT	56.11±2.11	76.39±12.34	20.00±4.97	30.98±4.71	20.00±4.97	92.22±8.19	0.561±0.023
	LR	64.44±4.67	82.33±12.08	37.78±6.69	51.35±6.86	37.78±6.69	91.11±6.92	0.685±0.068
	GLM	64.44±3.20	71.96±6.14	47.78±3.62	57.33±3.69	47.78±3.62	81.11±5.34	0.682±0.045
	NB	56.39±2.11	63.79±4.70	30.00±4.97	40.57±4.93	30.00±4.97	82.78±4.56	0.616±0.076
	RF	58.61±3.01	68.88±8.29	32.22±1.52	43.82±2.62	32.22±1.52	85.00±5.41	0.628±0.040
	GBT	70.00±2.11	67.00±2.98	79.44±4.21	72.59±1.50	79.44±4.21	60.58±6.63	0.794±0.009
	DL	65.00±2.06	64.51±1.74	66.67±3.93	65.54±2.52	66.67±3.93	63.33±2.32	0.734±0.065
	SVM	59.17±1.58	57.69±1.39	68.89±2.32	62.78±1.42	68.68±2.32	49.44±3.04	0.597±0.044
	FLM	NA	-	-	-	-	-	-
Db6	DT	54.44±2.67	62.73±7.23	20.56±6.09	30.77±7.81	20.56±6.09	88.33±1.24	0.587±0.026
	LR	60.00±1.16	60.62±1.43	57.22±3.17	58.83±1.75	57.22±3.17	62.78±3.17	0.652±0.041
	GLM	59.72±0.98	60.38±1.09	56.67±3.73	58.41±1.99	56.67±3.73	62.78±3.17	0.651±0.041
	NB	53.61±4.87	53.00±3.97	67.22±4.97	59.18±3.61	67.22±4.97	40.00±9.34	0.571±0.041

	RF	55.83±2.48	60.55±3.95	33.33±5.56	42.82±5.38	33.33±5.56	78.33±3.62	0.610±0.049
	GBT	78.33±0.76	75.89±2.01	83.33±5.20	79.31±1.53	83.33±5.20	73.33±4.21	0.866±0.029
	DL	70.83±2.60	68.98±2.89	76.11±5.76	72.24±2.76	76.11±5.76	65.56±5.76	0.808±0.039
	SVM	47.78±2.32	47.54±2.60	44.44±6.51	45.82±4.21	44.44±6.51	51.11±5.05	0.484±0.019
	FLM	49.72±2.67	49.46±3.87	32.78±6.33	39.24±5.44	32.78±6.33	66.67±5.20	0.484±0.065
Db8	DT	51.39±0.00	100.00±0.0	2.78±0.00	5.41±0.00	2.78±0.00	100.00±0.00	0.514±0.00
	LR	62.78±3.32	74.64±9.64	40.00±3.17	51.81±2.92	40.00±3.17	85.56±7.45	0.656±0.064
	GLM	67.50±3.04	79.71±6.07	47.22±5.56	59.11±4.86	47.22±5.56	87.78±4.21	0.702±0.046
	NB	57.78±3.20	56.96±3.21	65.00±2.48	60.65±1.93	65.00±2.48	50.56±7.19	0.564±0.067
	RF	52.78±2.60	51.52±1.42	95.00±2.32	66.80±1.68	95.00±2.32	10.56±3.62	0.543±0.061
	GBT	73.61±0.98	74.61±3.04	72.22±7.08	73.11±2.55	72.22±7.08	75.00±6.21	0.807±0.017
	DL	67.78±2.28	70.82±2.04	60.56±6.33	65.14±3.78	60.56±6.33	75.00±3.40	0.750±0.032
	SVM	59.44±3.46	57.85±3.02	70.00±3.04	63.33±2.83	70.00±3.04	48.89±5.05	0.609±0.069
	FLM	53.06±3.73	51.89±2.27	83.89±5.34	64.09±3.04	83.89±5.34	22.22±5.20	0.570±0.048

Table S3. Classification performance of the best ML models generated from the DWT-based processing of the ECG signals at a decomposition level of 3.

Wavelet	ML-model	Level-3						
		Accuracy	Precision	Recall	F-measure	Sensitivity	Specificity	AUC
Db2	DT	NA	-	-	-	-	-	-
	LR	65.28±2.20	77.26±3.79	43.33±3.17	55.48±3.24	43.33±3.17	87.22±2.48	0.703±0.057
	GLM	66.11±4.00	80.84±6.41	42.22±6.02	55.33±6.38	42.22±6.02	90.00±3.73	0.676±0.061
	NB	61.11±3.67	65.40±4.77	47.22±6.51	54.70±5.24	47.22±6.51	75.00±4.39	0.626±0.052
	RF	50.83±1.24	55.83±8.12	10.00±3.73	16.66±5.36	10.00±3.73	91.67±3.93	0.574±0.029
	GBT	72.50±3.73	77.44±2.19	63.33±7.71	69.54±5.29	63.33±7.71	81.67±1.52	0.810±0.048
	DL	60.00±2.48	69.87±5.74	35.56±3.62	47.00±3.69	35.56±3.62	84.44±4.21	0.687±0.024
	SVM	53.33±2.11	54.37±2.89	42.22±2.32	47.49±2.05	42.22±2.32	64.44±4.12	0.542±0.028
	FLM	56.67±1.81	54.89±1.44	75.56±4.97	63.51±1.74	75.56±4.97	37.78±6.39	0.573±0.031
Db4	DT	55.28±1.81	87.14±13.84	12.78±3.17	22.11±4.91	12.78±3.17	97.78±2.32	0.596±0.016
	LR	62.22±3.01	72.32±4.06	39.44±7.45	50.76±6.87	39.44±7.45	85.00±3.17	0.623±0.056
	GLM	67.78±4.21	76.34±7.79	52.22±2.32	61.92±3.78	52.22±2.32	83.33±7.08	0.691±0.038
	NB	60.83±3.32	0.602±0.065	66.44±6.15	44.44±5.56	53.05±4.68	44.44±5.56	77.22±6.02
	RF	50.56±2.52	50.42±1.86	69.44±3.93	58.39±2.23	69.44±3.93	31.67±4.65	0.548±0.026
	GBT	76.94±3.20	79.91±4.60	72.22±2.78	75.83±3.04	72.22±2.78	81.67±5.05	0.850±0.041
	DL	65.28±4.61	72.02±7.71	50.56±4.97	59.28±5.11	50.56±4.93	80.00±6.63	0.725±0.051
	SVM	64.17±2.48	67.22±3.55	55.56±1.96	60.81±2.27	55.58±1.96	72.78±4.12	0.682±0.021
	FLM	53.61±1.58	52.26±1.05	83.89±3.62	64.38±1.39	83.89±3.62	23.33±4.21	0.562±0.042
Db6	DT	52.22±1.58	51.16±0.83	97.78±2.32	67.17±1.22	97.78±2.32	6.67±1.52	0.532±0.062
	LR	63.89±2.60	72.27±6.72	46.11±4.21	56.03±3.01	46.11±4.21	81.67±6.69	0.707±0.034
	GLM	65.00±1.81	86.55±4.59	35.56±2.32	50.38±2.81	35.56±2.32	94.44±1.96	0.663±0.038
	NB	56.94±1.70	61.52±4.32	38.33±5.34	46.94±3.33	38.33±5.34	75.56±6.63	0.571±0.026
	RF	50.28±1.16	50.14±0.61	97.78±2.32	66.29±0.85	97.78±2.32	2.78±2.78	0.459±0.039
	GBT	75.83±3.49	75.18±5.09	77.78±3.93	76.33±2.90	77.78±3.93	73.89±7.24	0.839±0.033
	DL	64.17±3.17	71.02±3.53	47.78±6.02	57.01±4.90	47.78±6.02	80.56±2.78	0.726±0.010
	SVM	58.89±3.20	59.28±4.03	58.89±4.56	58.88±1.94	58.89±4.56	58.89±9.50	0.629±0.037
	FLM	40.17±1.58	28.57±0.00	1.11±1.52	2.14±0.00	-	97.22±2.78	0.534±0.040
Db8	DT	NA	-	-	-	-	-	-

	LR	58.61±2.28	66.08±5.67	36.11±1.96	46.60±1.95	36.11±1.96	81.11±4.97	0.623±0.037
	GLM	60.56±0.76	78.57±4.37	29.44±4.21	42.59±3.72	29.44±4.21	91.67±3.40	0.680±0.036
	NB	59.17±2.88	58.81±2.68	61.11±4.39	59.91±3.26	61.11±4.39	57.22±3.17	0.592±0.041
	RF	50.00±2.95	50.06±2.91	52.78±2.78	51.36±2.49	52.78±2.78	47.22±4.81	0.533±0.025
	GBT	73.06±2.11	87.62±3.76	53.89±5.05	66.56±3.62	53.89±5.05	92.22±3.04	0.817±0.030
	DL	64.72±2.11	87.68±4.08	34.44±5.41	49.20±5.34	34.44±5.41	95.00±2.32	0.769±0.037
	SVM	53.06±1.16	53.77±1.07	44.44±3.40	48.58±1.81	44.44±3.40	61.67±4.56	0.550±0.036
	FLM	54.72±3.75	54.00±3.48	66.11±2.32	59.39±2.36	66.11±2.32	43.33±7.51	0.564±0.049

Table S4. Classification performance of the best ML models generated from the DWT-based processing of the ECG signals at a decomposition level of 4.

Wavelet	ML-model	Level-4						
		Accuracy	Precision	Recall	F-measure	Sensitivity	Specificity	AUC
Db2	DT	NA	-	-	-	-	-	-
	LR	66.94±5.33	75.67±7.40	50.00±7.08	60.09±6.86	50.00±7.08	83.89±5.34	0.700±0.067
	GLM	64.44±2.71	80.93±8.37	38.33±3.04	51.87±3.31	38.33±3.04	90.56±5.05	0.699±0.063
	NB	59.17±3.62	63.39±6.01	43.89±4.97	51.74±4.73	43.89±4.97	74.44±5.69	0.627±0.034
	RF	58.06±2.06	75.83±8.13	23.89±3.17	36.22±3.96	23.89±3.17	92.22±3.04	0.602±0.060
	GBT	72.50±3.73	77.44±2.19	63.33±7.71	69.54±5.29	63.33±7.71	81.67±1.52	0.810±0.048
	DL	60.00±2.48	69.87±5.74	35.56±3.62	47.00±3.69	35.56±3.62	84.44±4.21	0.687±0.024
	SVM	63.61±3.60	65.61±3.67	57.22±6.97	60.98±5.04	57.22±6.97	70.00±4.97	0.683±0.053
	FLM	56.67±1.16	54.45±0.72	81.67±5.41	65.28±1.98	81.67±5.41	31.67±5.41	0.589±0.020
Db4	DT	NA	-	-	-	-	-	-
	LR	60.00±3.32	65.44±4.32	42.22±7.19	51.11±6.18	42.22±7.19	77.78±3.93	0.632±0.042
	GLM	53.33±2.11	65.24±8.09	14.44±4.56	23.38±6.42	14.44±4.56	92.22±3.62	0.650±0.095
	NB	58.89±2.11	62.29±2.65	45.00±3.62	52.21±3.13	45.00±3.62	72.78±2.32	0.605±0.033
	RF	48.61±3.26	48.31±3.64	44.44±7.61	46.15±5.65	44.44±7.61	52.78±5.56	0.540±0.050
	GBT	72.50±3.85	82.28±6.84	57.78±5.34	67.71±4.67	57.78±5.34	87.22±6.09	0.817±0.053
	DL	62.50±2.78	81.09±4.87	32.78±6.33	46.36±6.58	32.78±6.33	92.22±3.04	0.715±0.041
	SVM	59.44±3.73	61.86±4.83	49.44±3.62	54.94±4.01	49.44±3.62	69.44±4.39	0.607±0.016
	FLM	51.39±3.80	50.94±2.90	65.56±8.24	57.26±4.94	65.56±8.24	37.22±3.17	0.528±0.042
Db6	DT	56.11±4.46	66.54±10.62	25.56±8.43	36.26±9.75	25.56±8.43	86.67±6.63	0.591±0.067
	LR	61.67±4.12	59.25±2.86	73.89±9.74	65.64±5.29	73.89±9.74	49.44±4.56	0.701±0.707
	GLM	57.78±2.32	78.02±8.56	21.67±3.04	33.87±4.34	21.67±3.04	93.89±2.32	0.683±0.053
	NB	59.17±2.11	55.88±1.55	87.78±2.48	68.26±1.08	87.78±2.48	30.56±5.56	0.611±0.037
	RF	50.83±1.58	52.34±7.82	11.11±5.56	18.00±7.98	11.11±5.56	90.56±3.17	0.606±0.058
	GBT	69.72±3.85	72.54±4.77	63.89±8.56	67.65±5.33	63.89±8.56	75.56±6.33	0.778±0.034
	DL	62.22±6.09	63.48±6.43	57.22±7.51	60.15±6.90	57.22±7.51	67.22±5.34	0.680±0.036
	SVM	53.33±3.75	52.92±3.19	63.33±5.34	57.54±3.06	63.33±5.34	43.33±8.47	0.528±0.051
	FLM	51.67±4.33	52.22±6.34	38.33±7.19	44.04±6.49	38.33±7.19	65.00±6.09	0.522±0.049
Db8	DT	NA	-	-	-	-	-	-
	LR	53.61±2.32	65.67±9.40	13.89±5.56	22.68±8.23	13.89±5.56	93.33±1.52	0.620±0.069
	GLM	55.56±1.39	61.81±6.44	31.67±5.76	41.32±4.56	31.67±5.76	79.44±7.24	0.639±0.052
	NB	56.67±5.93	60.20±9.34	41.11±3.62	48.81±5.60	41.11±3.62	72.22±8.33	0.600±0.050
	RF	NA	-	-	-	-	-	-

	GBT	61.39±1.81	58.95±1.75	75.56±5.34	66.14±1.91	75.56±5.34	47.22±6.51	0.650±0.049
	DL	64.44±3.34	64.38±3.66	65.00±4.65	64.62±3.35	65.00±4.65	63.89±5.20	0.695±0.051
	SVM	56.11±1.56	66.32±4.44	25.00±1.96	36.27±2.44	25.00±1.96	87.22±2.48	0.606±0.044
	FLM	50.00±3.40	49.83±12.51	12.78±4.21	20.25±6.25	12.78±4.21	87.22±4.21	0.569±0.067

Table S5. Classification performance of the best ML models generated from the DWT-based processing of the ECG signals at a decomposition level of 5.

Wavelet	ML-model	Level-5						
		Accuracy	Precision	Recall	F-measure	Sensitivity	Specificity	AUC
Db2	DT	53.61±1.24	87.00±18.57	10.00±4.21	17.40±5.90	10.00±4.21	97.22±4.81	0.554±0.020
	LR	60.00±2.67	57.90±1.89	73.33±6.39	64.63±3.13	73.33±6.39	46.67±4.97	0.643±0.020
	GLM	58.33±4.39	60.58±6.23	48.89±3.73	54.02±4.11	48.89±3.73	67.78±7.24	0.641±0.047
	NB	56.67±3.01	58.42±3.75	46.11±4.65	51.49±4.13	46.11±4.65	67.22±3.62	0.638±0.038
	RF	51.94±2.71	51.03±1.44	96.11±3.17	66.66±1.98	96.11±3.17	7.78±2.32	0.617±0.062
	GBT	70.00±1.58	71.65±3.40	66.67±3.40	68.96±1.24	66.67±3.40	73.33±5.41	0.771±0.032
	DL	64.17±1.52	62.60±2.46	71.11±4.21	66.47±1.08	71.11±4.21	57.22±6.39	0.682±0.027
	SVM	55.66±3.11	53.90±2.15	76.67±6.97	63.22±3.35	76.67±6.97	34.44±6.09	0.573±0.050
	FLM	56.39±3.20	54.40±2.32	80.00±2.32	64.74±2.08	80.00±2.32	32.78±5.69	0.595±0.025
Db4	DT	49.44±3.62	47.25±11.22	17.22±6.63	25.08±8.60	17.22±6.63	81.67**±3.17**	0.475±0.058
	LR	58.33±2.60	67.73±6.20	32.22±4.21	43.52±4.34	32.22±4.21	84.44±4.21	0.660±0.039
	GLM	61.11±2.60	71.12±3.83	37.22±4.65	48.80±4.78	37.22±4.65	85.00±1.52	0.658±0.044
	NB	58.33±4.91	56.62±3.77	70.00±8.19	62.56±5.50	70.00±8.19	46.67±3.04	0.602±0.052
	RF	61.94±2.52	64.74±3.24	52.78±4.39	58.05±3.16	52.78±4.39	71.11±4.65	0.660±0.060
	GBT	71.11±0.62	74.18±2.16	65.00±3.17	69.21±1.05	65.00±3.17	77.22±3.62	0.773±0.023
	DL	65.56±2.67	69.57±3.33	55.56±5.89	61.62±4.00	55.56±5.89	75.56±4.97	0.694±0.040
	SVM	59.72±2.78	58.95±2.76	64.44±2.32	61.55±2.24	64.44±2.32	55.00±4.56	0.628±0.047
	FLM	56.11±4.46	57.74±5.56	46.11±5.76	51.17±5.26	46.11±5.76	66.11±6.33	0.598±0.037
Db6	DT	50.28±1.52	50.14±0.78	07.22±1.96	66.16±1.04	07.22±1.96	3.33±2.32	0.530±0.053
	LR	61.94±3.34	73.80±8.54	37.78±5.05	49.72±4.90	37.78±5.05	86.11±5.56	0.700±0.056
	GLM	60.83±1.81	66.77±2.79	43.33±4.65	52.42±3.64	43.33±4.65	78.33±3.62	0.642±0.030
	NB	57.78±2.71	60.91±4.35	44.44±1.96	51.30±1.79	44.44±1.96	71.11±6.09	0.643±0.062
	RF	57.50±1.24	61.57±1.41	40.00±5.05	48.34±3.74	40.00±5.05	75.00±3.40	0.584±0.036
	GBT	68.89±3.04	69.09±2.53	68.33±6.69	68.60±4.09	68.33±6.69	69.44±3.40	0.776±0.041
	DL	65.56±3.01	65.50±2.13	65.56±7.51	65.40±4.32	65.56±7.51	65.56±3.17	0.721±0.019
	SVM	61.39±2.28	61.32±1.96	61.67±4.97	61.43±3.03	61.67±4.97	61.11±3.40	0.641±0.038
	FLM	58.33±2.78	56.44±2.04	72.78±5.34	63.54±3.14	72.78±5.34	43.89±3.62	0.610±0.022
Db8	DT	51.11±3.32	50.60**±1.77**	93.89±4.12	65.75±2.40	93.89±4.12	8.33±3.93	0.585±0.072
	LR	53.89±3.60	59.68±9.16	22.78±5.69	32.88±7.25	22.78±5.69	85.00±2.48	0.626±0.042
	GLM	57.78±1.86	65.57±4.98	33.33±3.40	44.05±2.94	33.33±3.40	82.22±4.21	0.640±0.031
	NB	60.28±3.75	65.79±7.44	44.44±1.96	52.87±2.40	44.44±1.96	76.11±8.00	0.653±0.030
	RF	52.78±1.96	51.49±1.06	96.11±3.17	67.05±1.49	96.11±3.17	9.44±3.17	0.555±0.043
	GBT	66.67±2.41	68.15±3.99	63.33±6.33	65.42±3.16	63.33±6.33	70.00±6.63	0.752±0.023
	DL	65.28±2.60	64.08±4.08	71.11±8.91	67.03±3.30	71.11±8.91	59.44±9.74	0.700±0.014
	SVM	51.39±3.93	51.19±3.29	65.56±8.47	57.28±4.12	65.56±8.47	37.22±10.13	0.517±0.035
	FLM	52.50±2.85	55.61±6.19	26.67±4.65	35.80±4.87	26.67±4.65	78.33±6.02	0.570±0.033

3. WPD

Table S6. Classification performance of the best ML models generated from the WPD-based processing of the ECG signals at a decomposition level of 2.

Wavelet	ML-model	Level-2						
		Accuracy	Precision	Recall	F-measure	Sensitivity	Specificity	AUC
Db2	DT	50.00±0.00	50.00±00	100.0±0.0	50.00±0.0	100.0±0.0	100.0±0.0	0.572±0.037
	LR	59.72±1.96	77.39±5.93	27.78±3.93	40.70±4.37	27.78±3.93	91.67±3.40	0.636±0.068
	GLM	63.61±3.73	72.85±7.69	43.89±3.04	54.70±4.03	43.89±3.04	83.33±5.56	0.687±0.078
	NB	60.00±4.33	66.56±7.47	40.56±4.21	50.35±5.09	40.56±4.21	79.44±5.41	0.622±0.032
	RF	55.00±2.11	61.77±6.04	27.22±4.12	37.57±3.88	27.22±4.12	82.78±5.34	0.607±0.041
	GBT	71.11±4.21	69.37±3.76	75.56±4.56	72.22±4.12	75.56±4.56	66.67±3.93	0.794±0.053
	DL	64.44±3.75	69.53±5.06	51.67±5.41	59.17±4.59	51.67±5.41	77.22±4.97	0.747±0.030
	SVM	48.06±3.20	46.72±4.56	25.56±6.92	32.53±6.70	25.56±6.92	70.56±9.94	0.445±0.031
	FLM	51.39±3.26	52.07±4.56	42.22±4.56	46.43±2.75	42.22±4.56	60.56±8.87	0.547±0.036
Db4	DT	58.06±2.28	58.74±2.72	54.44±6.09	56.37±3.58	54.44±6.09	61.67±5.34	0.592±0.046
	LR	66.11±3.62	76.36±4.63	47.78±6.63	58.33±5.88	47.78±6.63	84.44±3.17	0.695±0.042
	GLM	62.78±5.84	67.85±7.26	48.33±7.51	56.38±7.37	48.33±7.51	77.22±5.34	0.696±0.076
	NB	57.22±3.46	60.75±5.56	40.56±8.24	48.33±6.98	40.56±8.24	73.89±5.76	0.591±0.061
	RF	52.22±0.76	57.64±2.95	17.22±4.97	26.19±5.55	17.22±4.97	87.22±4.21	0.562±0.039
	GBT	69.17±2.67	74.92±3.85	57.78±4.56	65.15±3.58	57.78±4.56	80.56±3.93	0.774±0.032
	DL	64.44±3.49	71.54±6.23	48.89±6.39	57.75±4.90	48.89±6.39	80.00±7.19	0.744±0.035
	SVM	53.06±3.85	56.23±7.65	27.78±6.51	36.94±6.85	27.78±6.51	78.33±5.34	0.526±0.043
	FLM	50.00±0.00	50.00±00	100.0±0.0	50.00±0.0	100.0±0.0	100.0±0.0	0.572±0.037
Db6	DT	50.00±0.00	50.00±00	100.0±0.0	50.00±0.0	100.0±0.0	100.0±0.0	0.572±0.037
	LR	64.72±2.52	80.66±2.92	38.89±6.80	52.15±6.08	38.89±6.80	90.56±2.48	0.720±0.060
	GLM	63.89±3.26	75.81±4.17	40.56±5.76	52.75±5.79	40.56±5.76	87.22±1.52	0.698±0.046
	NB	57.50±2.52	62.63±3.95	37.22±5.76	46.51±5.03	37.22±5.76	77.78±3.93	0.624±0.047
	RF	50.00±0.00	50.00±00	100.0±0.0	50.00±0.0	100.0±0.0	100.0±0.0	0.572±0.037
	GBT	67.78±5.93	70.59±7.51	61.11±6.51	65.45±6.62	61.11±6.51	74.44±6.63	0.715±0.064
	DL	66.39±3.01	67.68±3.27	62.78±4.21	65.10±3.41	62.78±4.21	70.00±3.62	0.750±0.045
	SVM	51.94±5.52	52.14±6.17	48.33±5.76	50.13±5.77	48.33±5.76	55.56±6.51	0.525±0.042
	FLM	51.39±3.26	52.07±4.56	42.22±4.56	46.43±2.75	42.22±4.56	60.56±8.87	0.547±0.036
Db8	DT	50.00±0.98	50.00±0.05	97.78±2.32	66.16±0.82	97.78±2.32	2.22±2.32	0.457±0.020
	LR	62.50±0.00	75.61±3.24	37.22±3.17	49.74±2.11	37.22±3.17	87.78±3.17	0.655±0.058
	GLM	58.61±2.28	90.56±10.32	19.44±3.93	31.83±5.58	19.44±3.93	97.78±2.32	0.682±0.062
	NB	52.22±1.86	51.22±1.01	92.78±3.17	66.00±1.62	92.78±3.17	11.67±1.24	0.508±0.026
	RF	57.22±3.85	55.27±2.61	75.56±8.19	63.73±4.18	75.56±8.19	38.89±6.80	0.578±0.075
	GBT	71.67±1.58	67.12±1.35	85.00±3.17	74.99±1.59	85.00±3.17	58.33±2.78	0.828±0.025
	DL	69.72±2.28	66.18±3.17	74.44±4.97	71.05±2.32	74.44±4.97	65.00±5.76	0.796±0.028
	SVM	61.67±1.58	61.41±2.14	63.33±4.97	62.23±2.23	63.33±4.97	60.00±5.41	0.649±0.026
	FLM	49.44±3.49	49.67±2.05	85.00±5.05	62.68±2.83	85.00±5.05	13.89±4.39	0.498±0.055

Table S7. Classification performance of the best ML models generated from the WPD-based processing of the ECG signals at a decomposition level of 3.

Wavelet	ML-model	Level-3						
		Accuracy	Precision	Recall	F-measure	Sensitivity	Specificity	AUC
Db2	DT	55.83±3.17	60.86±5.73	32.78±4.97	42.49±5.20	32.78±4.97	78.89±4.21	0.570±0.031
	LR	63.61±1.52	63.68±2.46	63.89±4.39	63.67±1.83	63.89±4.39	63.33±5.34	0.662±0.007
	GLM	63.89±2.60	61.66±2.37	73.89±5.41	67.13±2.61	73.89±5.41	53.89±6.09	0.715±0.027
	NB	54.17±2.78	53.36±2.29	66.67±3.40	59.25±2.45	66.7±3.40	41.67±4.39	0.578±0.024
	RF	53.33±1.58	51.75±0.83	98.33±2.48	67.81±1.23	98.33±2.48	8.33±1.96	0.583±0.052
	GBT	69.44±1.96	88.39±4.35	45.00±4.97	59.43±4.02	45.00±4.97	93.89±3.04	0.787±0.033
	DL	65.28±2.60	62.70±2.69	76.11±5.76	68.63±2.53	76.11±5.76	54.44±7.24	0.710±0.025
	SVM	53.06±2.48	62.41±10.39	17.22±2.32	26.79±2.88	17.22±2.32	88.89±5.56	0.578±0.063
	FLM	54.17±1.39	52.28±0.78	95.56±1.52	67.58±0.88	95.56±1.52	12.78±2.48	0.583±0.092
Db4	DT	NA	-	-	-	-	-	-
	LR	63.33±3.20	65.77±3.31	55.58±7.61	60.05±5.07	55.56±7.61	71.11±4.21	0.675±0.046
	GLM	58.89±3.20	57.49±2.85	68.89±3.62	62.63±2.57	68.89±3.62	48.89±6.09	0.650±0.046
	NB	55.83±2.28	55.67±2.63	58.89±5.34	57.08±2.28	58.89±5.34	57.78±7.61	0.571±0.005
	RF	52.50±2.48	51.38±1.38	92.78±3.17	66.13±1.89	92.78±3.17	12.22±2.48	0.572±0.081
	GBT	67.22±2.88	84.19±7.31	42.78±5.05	56.51±4.69	42.78±5.05	91.67±4.38	0.769±0.048
	DL	57.50±1.24	79.22±3.08	20.56±4.21	32.40±5.31	20.56±4.21	94.44±1.96	0.738±0.048
	SVM	NA	-	-	-	-	-	-
	FLM	53.61±2.32	52.67±1.73	71.11±3.17	60.51±2.16	71.11±3.19	36.11±2.78	0.549±0.041
Db6	DT	52.22±3.34	53.08±4.80	36.67±4.97	43.34±5.02	36.67±4.97	67.78±2.48	0.527±0.029
	LR	61.67±2.11	78.07±6.45	33.33±7.08	46.12±6.58	33.33±7.08	90.00±5.76	0.656±0.054
	GLM	60.28±3.75	58.00±2.98	75.00±2.78	65.40±2.83	75.00±2.78	45.56±5.41	0.665±0.063
	NB	53.89±2.67	53.48±2.35	58.89±7.19	55.93±4.20	58.89±7.19	48.89±5.05	0.567±0.020
	RF	51.11±3.60	50.96±3.78	49.44±6.92	50.14±5.37	49.44±6.02	52.78±1.96	0.548±0.048
	GBT	69.44±3.80	70.82±5.09	66.67±1.96	68.63±3.10	66.67±1.96	72.22±6.51	0.786±0.050
	DL	65.28±2.78	78.41±5.11	42.22±3.62	54.84±3.91	42.22±3.62	88.33±3.04	0.735±0.048
	SVM	55.00±2.11	52.79±1.19	95.00±3.62	67.85±1.60	95.00±3.62	15.00±4.21	0.555±0.023
	FLM	50.83±0.76	50.43±0.39	98.89±1.52	66.79±0.28	98.89±1.52	2.78±2.78	0.506±0.090
Db8	DT	55.00±2.11	52.79±1.19	95.00±3.62	67.85±1.60	95.00±3.62	15.00±4.21	0.555±0.023
	LR	61.11±2.60	74.77±5.99	33.89±6.02	46.33±5.81	33.89±6.02	88.33±4.56	0.667±0.060
	GLM	58.89±1.58	65.10±4.02	38.89±4.39	48.50±3.23	38.89±4.39	78.89±4.65	0.619±0.039
	NB	56.67±2.48	59.03±3.09	43.33±6.30	49.82±4.86	43.33±6.39	70.00±4.12	0.592±0.049
	RF	49.41±2.11	49.40±1.40	72.78±4.56	58.84±2.45	72.78±4.56	25.56±1.24	0.451±0.040
	GBT	66.94±4.75	65.92±4.67	70.56±6.09	68.07±4.55	70.56±6.09	63.33±6.63	0.733±0.047
	DL	60.28±3.20	57.43±2.35	79.44±3.73	66.66±2.76	79.44±3.73	41.11±3.62	0.685±0.066
	SVM	48.61±4.05	48.82±2.82	67.78±9.94	56.66±5.30	67.78±9.94	29.44±4.21	0.488±0.074
	FLM	55.00±1.86	53.00±1.08	88.33±5.34	66.21±2.03	88.33±5.34	21.67±4.56	0.585±0.045

Table S8. Classification performance of the best ML models generated from the WPD-based processing of the ECG signals at a decomposition level of 4.

Wavelet	ML-model	Level-4						
		Accuracy	Precision	Recall	F-measure	Sensitivity	Specificity	AUC
Db2	DT	53.61±3.34	54.48±4.13	43.89±5.34	48.53±4.52	43.89±5.34	63.33±4.97	0.545±0.045
	LR	63.33±4.24	65.03±5.62	58.89±3.04	61.68±3.17	58.89±3.04	67.78±8.24	0.649±0.050
	GLM	56.94±3.54	67.85±10.15	27.22±5.34	38.58±5.90	27.22±5.34	86.67±5.34	0.652±0.053
	NB	59.17±3.62	60.41±3.45	52.78±6.51	56.26±4.92	52.78±6.51	65.56±3.17	0.036±0.040
	RF	58.06±3.01	59.11±3.48	52.78±4.39	55.68±3.28	52.78±4.39	63.33±5.34	0.592±0.075
	GBT	73.33±1.16	73.13±3.02	74.44±7.45	73.50±2.50	74.44±7.45	72.27±6.51	0.795±0.046
	DL	64.44±2.32	76.56±3.91	41.67±3.40	53.92±3.47	41.67±3.40	87.22±2.48	0.730±0.026
	SVM	57.50±2.11	89.44±10.83	17.22±3.62	28.72±5.16	17.22±3.62	97.78±2.32	0.697±0.054
	FLM	53.06±2.85	58.41±7.93	21.11±6.39	30.68±7.25	21.11±6.39	85.00±4.21	0.586±0.026
Db4	DT	NA	-	--	-	-	-	-
	LR	58.89±0.76	66.95±2.62	35.56±4.12	46.26±2.96	35.56±4.12	82.22±4.21	0.645±0.034
	GLM	56.94±2.60	63.77±4.72	32.22±4.21	42.72±4.40	32.22±4.21	81.67±3.17	0.598±0.039
	NB	58.33±0.98	58.57±0.75	57.22±7.24	57.66±3.90	57.22±7.24	59.44±5.76	0.567±0.025
	RF	55.83±3.32	54.05±2.39	78.33±6.02	63.90±3.06	78.33±6.02	33.33±6.51	0.598±0.061
	GBT	66.11±4.24	65.77±4.01	67.22±6.92	66.39±4.78	67.22±6.92	65.00±5.05	0.730±0.027
	DL	62.22±4.85	66.97±7.24	48.89±5.76	56.37±5.60	48.89±5.76	75.56±7.19	0.692±0.061
	SVM	NA	-	-	-	-	-	-
	FLM	53.06±3.85	51.81±2.29	89.44±2.32	65.60±2.38	89.44±2.32	16.67±5.89	0.597±0.045
Db6	DT	50.00±2.60	49.99±1.87	70.00±5.69	58.27±3.12	70.00±5.69	30.00±3.62	0.486±0.050
	LR	57.22±5.14	68.99±12.89	25.56±6.63	37.24±8.89	25.56±6.63	88.89±3.93	0.658±0.065
	GLM	58.61±6.01	66.32±9.90	33.89±8.65	44.74±9.71	33.89±8.65	83.33±3.93	0.659±0.061
	NB	59.27±2.60	59.57±2.40	60.56±6.02	59.96±3.59	60.56±6.02	58.89±4.56	0.621±0.044
	RF	54.72±4.56	50.07±5.68	45.56±8.91	49.89±6.02	45.56±8.91	83.89±9.00	0.564±0.053
	GBT	68.61±3.75	76.72±7.07	53.89±2.48	63.25±3.69	53.89±2.48	83.33±5.89	0.724±0.052
	DL	58.89±5.34	60.07±6.73	54.44±5.05	57.01±4.99	54.44±5.05	63.33±8.43	0.632±0.047
	SVM	51.94±2.71	51.48±2.05	68.33±4.21	58.69±2.50	68.33±4.21	35.56±4.56	0.542±0.045
	FLM	58.06±3.98	55.93±3.10	77.22±4.97	64.80±2.97	77.22±4.97	38.89±8.10	0.605±0.043
Db8	DT	NA	-	-	-	-	-	-
	LR	54.17±2.20	53.57±1.87	61.67±9.09	57.13±4.48	61.67±9.09	46.67±6.92	0.577±0.042
	GLM	55.83±1.52	57.59±1.97	44.44±5.20	50.03±3.49	44.44±5.20	67.22±4.50	0.599±0.019
	NB	57.50±2.32	57.55±2.30	57.78±6.02	57.51±3.26	57.78±6.02	57.22±6.39	0.594±0.022
	RF	50.28±4.85	50.22±3.40	71.67±4.56	59.05±3.83	71.67±4.56	28.89±5.76	0.522±0.049
	GBT	62.22±2.28	59.47±1.99	77.22±3.04	67.15±1.65	77.22±3.04	47.22±5.20	59.47±1.99
	DL	57.78±2.32	63.16±3.64	37.22±3.73	46.80±3.74	37.22±3.73	78.33±2.32	0.635±0.023
	SVM	53.33±1.58	51.77±0.85	98.33±1.52	67.82±0.84	98.33±1.52	8.33±3.40	0.631±0.040
	FLM	50.00±0.00	50.03±3.45	25.00±3.93	33.22±3.79	25.00±3.93	75.00±3.93	0.571±0.029

Table S9. Classification performance of the best ML models generated from the WPD-based processing of the ECG signals at a decomposition level of 5

Wavelet	ML-model	Level-5						
		Accuracy	Precision	Recall	F-measure	Sensitivity	Specificity	AUC

Db2	DT	52.56±3.60	52.78±3.89	50.0±5.59	51.21±3.89	50.00±5.56	55.00±7.71	0.553±0.046
	LR	56.11±2.11	68.40±8.28	23.33±3.17	34.64±3.64	23.33±3.17	88.89±3.93	0.604±0.022
	GLM	57.22±3.01	64.62±7.35	32.78±4.12	43.32±4.13	32.78±4.12	81.67±5.41	0.637±0.058
	NB	61.11±2.95	62.13±2.89	56.67±5.05	59.23±3.93	56.67±5.05	65.56±2.48	0.645±0.037
	RF	58.89±2.88	58.68±2.76	60.00±5.76	59.26±3.67	60.00±5.76	57.78±4.12	0.611±0.019
	GBT	63.61±2.48	67.50±4.60	53.33±4.12	59.41±2.53	53.33±4.12	73.89±6.39	0.698±0.032
	DL	62.50±3.62	62.28±3.97	60.00±6.39	61.44±4.30	60.00±6.39	65.00±6.39	0.676±0.041
	SVM	NA	-	-	-	-	-	-
	FLM	58.33±0.98	56.26±0.74	75.00±4.39	64.25±1.66	75.00±4.39	41.67±3.93	0.621±0026
Db4	DT	50.00±0.00	50.00±0.00	100.00±0.00	66.67±0.00	100.00±0.00	0.00±0.00	0.421±0.058
	LR	58.61±3.85	59.71±5.45	55.56±3.93	57.33±2.30	55.56±3.93	61.67±9.90	0.596±0.037
	GLM	60.56±2.11	59.56±2.19	66.11±2.32	62.63±1.68	66.11±2.32	55.00±4.12	0.653±0.019
	NB	59.72±2.41	59.62±2.59	60.58±4.12	60.02±2.63	60.56±4.12	58.89±4.56	0.623±0.044
	RF	51.39±0.98	50.72±0.51	97.78±2.32	66.79±0.69	97.78±2.32	5.00±3.04	0.542±0.020
	GBT	57.22±1.16	55.29±0.84	75.56±3.04	63.83±1.33	75.56±3.04	38.89±2.78	0.648±0.019
	DL	64.17±3.85	64.12±4.01	64.44±3.62	64.27±3.74	64.44±3.62	63.89±4.39	0.679±0.050
	SVM	NA						
	FLM	56.67±1.16	55.28±1.00	70.00±2.32	61.76±1.05	70.00±2.32	43.33±3.17	0.606±0.037
Db6	DT	46.67±2.32	47.94±1.45	78.33±4.12	59.47±2.24	78.33±4.12	15.00±2.48	0.489±0.024
	LR	54.72±2.11	65.61±4.33	19.44±5.89	29.70±7.19	19.44±5.89	90.00±2.48	0.604±0.024
	GLM	55.28±4.75	68.32±15.41	21.67±6.02	32.42±7.95	21.67±6.02	88.89±7.08	0.616±0.041
	NB	60.00±4.21	60.40±3.95	57.22±8.24	58.66±6.00	57.22±8.24	62.78±3.17	0.623±0.032
	RF	50.56±1.24	50.37±0.76	82.22±3.73	62.43±0.62	82.22±3.73	18.89±6.02	0.545±0.035
	GBT	61.39±4.33	70.25±9.33	40.56±3.17	51.28±4.35	40.56±3.17	82.22±7.24	0.696±0.064
	DL	54.17±4.71	54.14±4.43	56.67±6.09	55.24±4.32	56.67±6.09	51.67±8.47	0.589±0.059
	SVM	NA						
	FLM	57.78±3.62	58.42±4.14	54.44±6.39	56.21±4.59	54.44±6.39	61.11±6.51	0.623±0.063
Db8	DT	53.33±6.10	53.19±5.95	52.22±9.50	52.59±7.65	52.22±9.50	54.44±6.09	0.545±0.067
	LR	54.72±2.11	54.16±1.91	61.67±6.33	57.55±3.23	61.67±6.33	47.78±6.02	0.562±0.045
	GLM	55.00±1.86	51.64±1.29	73.89±5.41	62.00±2.34	73.89±5.41	36.11±5.20	0.615±0.023
	NB	60.00±1.81	60.59±1.48	57.22±6.69	58.70±3.81	57.22±6.69	67.78±4.65	0.636±0.034
	RF	54.44±1.52	52.53±0.87	92.22±4.12	66.91±1.48	92.22±4.12	16.67±3.93	0.577±0.077
	GBT	60.28±3.04	59.30±3.48	67.22±3.62	62.67±1.50	67.22±3.62	53.33±8.65	0.664±0.026
	DL	63.06±3.34	66.29±2.54	52.78±9.42	58.48±6.44	52.78±9.42	73.33±3.73	0.650±0.054
	SVM	48.89±2.06	33.33±0.00	2.22±4.97	4.17±0.00	2.22±4.97	95.56±3.73	0.554±0.095
	FLM	57.78±2.52	55.36±1.97	81.11±3.62	65.76±1.78	81.11±3.62	34.44±5.76	0.590±0.055

4. Performance with all feature

Table S10: Classification performance of the best two ML models, after feeding all extracted features in each decomposition method individually and simultaneously.

		Accuracy	Precision	Recall	F-measure	Sensitivity	Specificity	AUC
EMD	NB	50.8±4.3	50.8±3.7	57.8±5.3	54.0±3.7	57.8±5.3	43.9±8.0	0.527±0.038
	GLM	53.6±2.1	52.8±1.5	67.8±5.0	59.3±2.7	67.8±5.0	39.4±3.6	0.573±0.021
	LR	52.8±2.0	51.5±1.0	93.3±4.2	66.4±1.8	93.3±4.2	12.2±3.2	0.524±0.044
	FLM	47.5±2.1	48.5±1.3	80.6±4.4	60.5±2.2	80.6±4.4	14.4±1.2	0.497±0.051
	DL	56.9±2.2	57.2±2.9	56.7±4.6	56.8±2.2	56.7±4.6	57.2±6.7	0.576±0.038

	DT	51.4±1.4	__	3.9±3.2	_	3.9±3.2	98.9±1.5	0.514±0.014
	RF	49.7±1.8	48.2±5.7	13.3±7.2	20.2±9.2	13.3±7.2	86.1±6.2	0.586±0.081
	GBT	51.1±2.7	52.9±7.0	20.0±3.6	29.0±4.6	20.0±3.6	82.2±3.2	0.585±0.039
	SVM	50.00±0.0	50.0±0.0	100.0±0.0	66.7±0.0	100.0±0.0	0.0±0.0	0.500±0.000
DWT	NB	58.3±1.7	57.8±1.6	61.7±5.3	59.6±2.7	61.7±5.3	55.0±4.6	0.612±0.019
	GLM	63.9±2.0	77.7±5.9	39.4±3.6	52.1±3.1	39.4±3.6	88.3±4.6	0.727±0.04
	LR	50.0±0.0	_	0.0±0.0	__	0.0±0.0	100.0±0.0	0.500±0.00
	FLM	62.8±2.7	61.9±2.5	66.7±5.6	64.1±3.2	66.7±5.6	58.9±4.6	0.656±0.055
	DL	70.6±2.7	69.6±3.4	73.3±1.5	71.4±2.0	73.3±1.5	67.8±5.0	0.800±0.035
	DT	54.4±3.6	54.1±3.5	58.3±7.1	56.0±4.3	58.3±7.1	50.6±6.6	0.588±0.063
	RF	57.2±2.7	55.1±2.0	78.3±3.6	64.7±2.3	78.3±3.6	36.1±4.4	0.627±0.024
	GBT	76.9±2.7	84.5±2.6	66.1±6.0	74.0±3.8	66.1±6.0	87.8±2.5	0.859±0.041
	SVM	50.0±0.0	_	0.0±0.0	_	0.0±0.0	100.0±0.0	0.638±0.036
WPD	NB	60.6±3.2	62.1±4.1	55.0±5.3	58.2±3.4	55.0±5.3	66.1±7.2	0.611±0.046
	GLM	61.4±2.5	74.4±8.6	36.1±3.4	48.3±2.2	36.1	86.7±7.2	0.671±0.056
	LR	50.0±0.0	50.0±0.0	100.0±0.0	66.7±0.0	100.0±0.0	0.0±0.0	0.543±0.075
	FLM	56.4±2.9	66.8±9.2	26.1±6.1	37.2±6.7	26.1±6.1	86.7±5.3	0.648±0.041
	DL	68.3±6.1	69.5±6.9	65.6±5.4	67.5±6.1	65.6±5.4	71.1±7.0	0.759±0.049
	DT	52.2±3.2	51.3±1.9	87.2±3.2	64.6±2.2	87.2±3.2	17.2±4.6	0.604±0.056
	RF	54.4±2.7	52.5±1.6	95.0±3.6	67.6±1.4	95.0±3.6	13.9±7.1	0.658±0.054
	GBT	66.9±3.6	68.7±3.9	62.8±9.3	65.3±5.2	62.8±9.3	71.1±6.4	0.767±0.043
	SVM	50.00±0.00	_	0.0±0.0	_	0.0±0.0	100.0±0.0	0.500±0.00
EMD+DWT+WPD	NB	61.4±3.5	63.3±4.3	54.4±7.5	58.3±5.2	54.4±7.5	68.3±5.4	0.613±0.03
	GLM	61.1±2.6	79.3±12.3	31.1±3.6	44.4±3.7	31.1±3.6	91.1±5.3	0.679±0.058
	LR	50.0±0.0	_	0.0±0.0	__	0.0±0.0	100.0±0.0	0.500±0.000
	FLM	61.7±0.8	58.7±0.4	78.9±3.7	67.3±1.4	78.9±3.7	44.4±2.8	0.654±0.026
	DL	68.9±2.9	71.1±3.5	63.9±5.9	67.2±3.7	63.9±5.9	73.9±4.6	0.775±0.027
	DT	56.9±1.7	56.2±1.8	63.3±6.0	59.4±2.7	63.3±6.0	50.6±6.6	0.597±0.050
	RF	57.2±3.7	60.6±5.1	41.1±6.0	48.9±5.6	41.1±6.0	73.3±4.2	0.601±0.070
	GBT	69.7±4.9	66.3±3.9	80.0±6.6	72.5±4.8	80.0±6.6	59.4±4.6	0.790±0.045
	SVM	50.0±0.0	50.0±0.0	100.0±0.0	66.7±0.0	100.0±0.0	0.0±0.0	0.500±0.00
